# A novel construct with biomechanical flexibility for articular cartilage regeneration

**DOI:** 10.1186/s13287-019-1399-2

**Published:** 2019-09-23

**Authors:** Baixiang Cheng, Teng Tu, Xiao Shi, Yanzheng Liu, Ying Zhao, Yinhua Zhao, Yijie Li, Hui Chen, Yongjin Chen, Min Zhang

**Affiliations:** State Key Laboratory of Military Stomatology & National Clinical Research Center for Oral Diseases & Shaanxi International Joint Research Center for Oral Diseases, Department of General Dentistry and Emergency, School of Stomatology, Fourth Military Medical University, No. 145 West Changle Road, Xi’an, 710032 China

**Keywords:** Cartilage regeneration, BMSCs, PRF, Mechanobiology, Hydrostatic pressure

## Abstract

**Background:**

Although tissue-engineered cartilage has been broadly studied, complete integration of regenerated cartilage with residual cartilage is still difficult for the inferior mechanical and biochemical feature of neocartilage. Chondrogenesis of mesenchymal stem cells can be induced by biophysical and biochemical factors.

**Methods:**

In this study, autologous platelet-rich fibrin (PRF) membrane was used as a growth factor-rich scaffold that may facilitate differentiation of the transplanted bone marrow mesenchymal stem cells (BMSCs). At the same time, hydrostatic pressure was adopted for pre-adjustment of the seed cells before transplantation that may promote the mechanical flexibility of neocartilage.

**Results:**

An in vitro study showed that the feasible hydrostatic pressure stimulation substantially promoted the chondrogenic potential of in vitro-cultured BMSC/PRF construct. In vivo results revealed that at every time point, the newborn tissues were the most favorable in the pressure-pretreated BMSC/PRF transplant group. Besides, the transplantation of feasible hydrostatic pressure-pretreated construct by BMSC sheet fragments and PRF granules could obviously improve the integration between the regenerated cartilage and host cartilage milieu, and thereby achieve boundaryless repair between the neocartilage and residual host cartilage tissue in rabbit temporomandibular joints. It could be concluded that feasible hydrostatic pressure may effectively promote the proliferation and chondrogenic differentiation of BMSCs in a BMSC/PRF construct.

**Conclusion:**

This newly formed construct with biomechanical flexibility showed a superior capacity for cartilage regeneration by promoting the mechanical properties and integration of neocartilage.

**Electronic supplementary material:**

The online version of this article (10.1186/s13287-019-1399-2) contains supplementary material, which is available to authorized users.

## Background

Despite its seemingly simple structure of the articular cartilage, the avascular nature of cartilage compromises its endogeneous capacity for repair, leading to a high incidence of unsolved cartilage-related injuries. Once damaged, full recovery of its structure, function, and biomechanical properties is not expected in most cases, and the damaged articular cartilage can proceed toward degeneration [1]. Traditional techniques for cartilage repair include marrow stimulation, allografts, and autografts. Although successful in some aspects, each of these techniques has limitations, such as forming fibrocartilage of inferior quality that does not persist, lacks integration, or acquires additional defects, especially when the lesion is beyond a limited size and severity [2]. Next-generation cell-based therapy uses stem cells instead of chondrocytes [3, 4]. Bone marrow mesenchymal stem cells (BMSCs) are the most widely adopted seed cells for cartilage regeneration [5–7]. Preclinical and clinical trials using adult mesenchymal stem cells have demonstrated encouraging results, although there are many unsolved problems at this moment [1]. Regenerated cartilage often undergoes degeneration and reorganization into fibrocartilaginous tissue and may eventually disappear, leading to delamination and exposure of subchondral bone [8]. There is still a gap in the proteoglycan content and mechanical properties between the neocartilage and autologous articular cartilage [9, 10]. Till now, ideal physiological and functional cartilage regeneration has not been achieved [11, 12]. It was hypothesized that the lack of success was due to the lack of some critical factors and cues for chondrogenic cell differentiation and biomechanical adaption from the seed cells’ milieu.

For cartilage regeneration, the matrix milieu aims to provide the transplanted stem cells a stable chondrogenic differentiation environment, while the mechanical milieu is critical to the quality and quantity of neocartilage tissues [12, 13]. It was known that the biomechanical environment of the articular cartilage, consisting of forces over a large range of motion, can take a devastating toll on neocartilage that lacks adequate biomechanical properties [9]. Because stem cells have a greater mechanical sensitivity than adult cells [13], biomechanical signals play a key role in regulating the phenotypic differentiation of stem cells [14]. In fact, to repair articular cartilage defects, tissue-engineered cartilage transplant inevitably has to fulfill the function of a load-bearing structure. Therefore, the transplant must regenerate and then play its role in a particular mechanical milieu. Despite the critical role of mechanical cues in articular cartilage maintenance [15–17], higher repair efficiency, lateral integration, and further functional cartilage regeneration by biomechanical milieu regulation have not been implemented.

Here we set out to develop a novel scaffold-free tissue-engineered cartilage transplant with biomechanical flexibility that may show a superior capacity for cartilage regeneration. To achieve this, autologous platelet-rich fibrin (PRF) membrane was used as a growth factor-rich scaffold that may facilitate differentiation of the transplanted BMSCs. At the same time, hydrostatic pressure was adopted for pre-adjustment of the seed cells before transplantation that may promote the mechanical flexibility of neocartilage. Our results provide the first evidence that the transplantation of mechanically pretreated construct by BMSC sheets fragments and PRF granules could obviously improve the integration between the regenerated cartilage and host cartilage milieu, and thereby achieve boundaryless repair between the neocartilage and residual host cartilage tissue in the temporomandibular joint (TMJ). This work demonstrates that biomechanical pressure is an indispensable factor that promotes tissue-engineered regeneration and repair of TMJ articular cartilage and plays a crucial role in improving the mechanical properties and integration of neocartilage. It may provide valuable insights into the development of future treatment options for articular cartilage regenerative medicine.

## Methods

### Derivation of BMSC sheet and PRF membrane

Adult male New Zealand rabbits (approximately 4 months old) were obtained from the Animal Center, Fourth Military Medical University. BMSCs were cultured using the whole marrow-adherence method, and then were identified (see Additional file 1: Figure S1). The rabbit BMSCs (P3) obtained in the previous section were placed into six-well plates at a density of 3 × 10^4^ cells/well in complete medium until they reached subconfluence. Vitamin C was then added at a concentration of 48 μg/ml for a further incubation until the edge of the cell sheet became slightly rolled up, which is believed to be an indication of cell sheet maturation. For PRF preparation, 10-ml blood samples were extracted aseptically from the central auricular arteries of rabbits using a 10-ml disposable syringe and then immediately transferred into glass centrifuge tubes. PRF was obtained by immediate centrifugation of blood samples at 3000 r/min for 10 min. The PRF clot formed in the middle layer of the tube was taken using sterile tweezers and gently compressed with two pieces of sterile dry gauze for 10 s, to form the PRF membrane. The growth curve of BMSCs in the BMSC/PRF construct and continued growth factor release from PRF in the BMSC/PRF construct over 96 h were detected (see Additional file 2: Figure S2 and Additional file 3: Table S1).

### Selection of favorable hydrostatic pressure conditions for BMSC proliferation and chondrogenic differentiation in co-cultured BMSC/PRF construct

The indirectly co-cultured BMSC cell sheet and PRF membrane were stimulated with hydrostatic pressure using a custom-designed multi-functional in vitro cell compression system (see Additional file 4: Figure S3). The specific characteristics of the compression device, its operation procedures, and the selection of favorable pressure conditions are described [18–20]. Our group has demonstrated that in an environment loaded with pressure using the compression device, 90 kPa for 1 h can result in extensive biological effects on BMSCs, including enhanced cell proliferation activity, elevated alkaline phosphatase activity, upregulation of estrogen receptor-α, and assembly of intracellular stress fibers [21]. In the present study, we compressed the BMSC/PRF construct rather than monolayer-cultured BMSCs to investigate the effects of biomechanical stimulation to the chondrogenic characteristics of the construct. Therefore, according to the optimal pressure condition for BMSCs selected in the previous experiments, we increased the force value and prolonged the time to determine the most suitable conditions of pressure stimulation for chondrogenic differentiation of BMSCs in the construct. During the experiment, six-well plates containing the construct were placed in the cell compression device and stimulated with pressure under fixed conditions for either 1 or 6 h every day for 2, 4, or 6 days. Thus, the 2-day, 4-day, and 6-day treatment groups were each subdivided into 0 kPa/1 h, 0 kPa/6 h, 90 kPa/1 h, 90 kPa/6 h, 120 kPa/1 h, 120 kPa/6 h, 150 kPa/1 h, and 150 kPa/6 h subgroups.

For real-time PCR (polymerase chain reaction) analysis, the total cellular RNA was isolated from the maintenance cell cultures with Trizol (Invitrogen, Carlsbad, CA, USA) to evaluate the expression of cell proliferation markers, including *Pcna* (proliferating cell nuclear antigen), and chondrogenesis-related genes, including *Sox9* (SRY-related HMG box-9), *Col II* (collagen II), and *Acan* (Aggrecan) by real-time PCR (TaKaRa Bio, Tokyo, Japan). The reaction product was quantified using a relative quantification tool (CFX Manager, Bio-Rad) with *Gadph* as a reference gene. The primer sequences for *Pcna*, *Acan*, *Sox9*, and *Col II* (Sango Biotech, Shanghai, China) are listed in Additional file 5: Table S2. Afterwards, the effects of the hydrostatic stimulation of the BMSC/PRF construct on cartilage regeneration were evaluated both in vitro and in vivo.

### Electron microscopic observation of the pressure effects on the ultrastructure of BMSCs co-cultured with PRF

The BMSC sheet and indirectly co-cultured BMSC sheet and PRF treated with and without the abovementioned optimal mechanical stimulation were prepared to observe the ultrastructural changes of BMSCs. After treatment, the cell sheet was immediately fixed in 3% glutaraldehyde–1.5% paraformaldehyde followed by 1% osmic acid–1.5% potassium ferrocyanide and then observed using transmission electron microscopy (TEM). Additionally, the culture solution for each culture specimen was removed. The specimens were immersed in 2.5% glutaraldehyde and fixed at 4 °C for 2 h. After CO_2_ critical-point drying and gold-ion sputtering, the specimens were observed, and images were acquired by scanning electron microscopy (SEM).

### Construction of the BMSC/PRF tissue-engineering transplant

The rabbit BMSC sheet and PRF were prepared as previously described in “Derivation of BMSC sheet and PRF membrane”. Then, the PRFs were cut into small pieces (0.5 × 0.5 × 0.5 mm) in a sterile dish. The cell sheet was stripped with forceps from the dish and chopped into cell sheet fragments (0.5 × 0.5 mm). The transplanted BMSC/PRF construct for subsequent ectopic or orthotopic cartilage tissue regeneration was obtained by combining cell sheet fragments of BMSCs with PRF granules at the ratio that we selected prior to this work (see Additional file 6: Figure S4 and Additional file 7: Figure S5) [22].

### Ectopic transplantation of the biomechanical pressure-pretreated BMSC/PRF construct in nude mice

Forty-five nude mice (6-week-old males; Fourth Military Medical University Animal Center, Xi’an, China) were used. Mice were equally and randomly assigned to three groups for transplantation of BMSC sheet, BMSC/PRF construct, and pressure-pretreated BMSC/PRF construct, respectively. Anesthesia was achieved by intramuscular injection of 1% sodium pentobarbital (30 mg/kg). An approximately 1-cm skin incision was made aseptically above the posterior superior iliac spine along both sides of the back of each mouse. Blunt dissection of the subcutaneous tissue formed two subcutaneous pockets. Each group of animals was transplanted with a pure BMSC sheet, a statically cultured BMSC/PRF construct, or a pressure-pretreated BMSC/PRF construct. The material wrapping a 0.028-g fragment of auricular cartilage was transplanted into the subcutis of nude mice to imitate the cartilage milieu. Thereafter, the incision was closely sutured. Specimens were taken at 2, 4, and 8 weeks after surgery, five mice at each time point. The specimens were fixed in 4% paraformaldehyde for 48 h and then dehydrated, cleared, and embedded in paraffin following conventional procedures. Subsequently, the specimens were sliced longitudinally from the central area, and the sections were subjected to hematoxylin and eosin (HE) and toluidine blue (TB) staining before light microscopy observation.

Samples for glycosaminoglycan (GAG) content determination were taken at predetermined time points and lyophilized, and digested with 1 ml of 0.1% papain solution. Hoechst 33258 was added for DNA dye, and the absorbance was measured by a fluorescence spectrophotometer at 460 nm to detect the total DNA content in the sample. Dimethyl methylene blue was added, and the absorbance of each sample was detected at 525 nm to determine the content of GAG in the sample. The ratio of GAG content to DNA content (GAG/DNA) was characterized as GAG secreting ability of each sample. Six parallel samples were set up in the experiment.

### Animal surgery and groups

A total of 180 skeletally mature, male, 6-month-old New Zealand White rabbits, obtained from the Animal Center (Fourth Military Medical University), were used to create a full-thickness, bilateral chondral defect model based on previous studies [23, 24]. Animals were randomly assigned to five groups: blank control (natural recovery, Group A), PRF alone (Group B), BMSC sheet alone (Group C), BMSC/PRF construct (Group D), and pressure-pretreated BMSC/PRF construct (Group E). First, we established a rabbit model of articular cartilage defect in unilateral TMJ (3 mm in diameter and 3 mm in depth) (see Additional file 8: Figure S6). Then, animals of the control group were used to simply establish a condylar cartilage defect model, without any repair treatment. Animals of the PRF alone group were transplanted with the PRF membrane. The PRF was trimmed into small pieces slightly larger than the condylar cartilage defect area and carefully filled into the defect. The PRF membrane was kept in close contact with the hole wall, and its surface was level with the surface of the surrounding normal articular cartilage. The same procedure was followed to transplant the BMSC sheet, the BMSC/PRF construct, and the pressure-pretreated BMSC/PRF construct into the corresponding groups. Then, the articular disc was repositioned, and the skin and joint capsule were closed. To ensure normal eating after surgery, for all the experimental animals, only right TMJ received defect and repair operation, with the other side of TMJ receiving just open-and-close operation as a sham. Total repetition for each experimental group was 12 (*n* = 12). To minimize discomfort, carprofen (4 mg/kg) was administered for 3 days postoperatively. All animals were returned to their cages where they were allowed unrestricted weight-bearing activity and were observed for signs of pain, infection, and proper activity.

### Histomorphometric evaluation of explants

Animals were sacrificed 2, 4, or 8 weeks after surgery to collect specimens. For histological staining, after gross observation of the repaired tissue, the specimens were immediately fixed in 4% paraformaldehyde for 48 h and then dehydrated, cleared, and embedded in paraffin following conventional procedures. Sections were sliced along the sagittal plane of condyle, perpendicular to the defect repair area. The section specimens were stained with HE staining and 1% TB staining before light microscopy observation. For each sample, five slides were randomly selected and five high power fields (× 200) on each slice were randomly selected for chondrocyte-like cells counting, which appeared as round or oval cells by HE staining. Histological sections from each location of each joint were scored blindly and independently using a well-established scoring algorithm for osteochondral tissue repair. Seven total parameters were used to analyze tissue repair for the cartilage defect according to the modified histomorphometric evaluation of explants as the individual chondral and subchondral regions [25] (see Additional file 9: Table S3). The overall defect repair was evaluated for the percent filled with newly formed tissue and percent degradation of the implant. The chondral defect region was evaluated for tissue morphology, thickness, regularity, chondrocyte clustering, and cell/GAG content. The subchondral defect region was examined for quality of new tissue filling, integration, and cartilage morphology.

### Chondrogenetic gene detection in repaired cartilage

For genetic detection, specimens were collected and immediately placed into microcentrifuge tubes containing 1 ml of PCR preservative solution. The tubes were placed on ice, and cartilage was resected from the defect site in the condyle and fully ground in liquid nitrogen. For real-time PCR analysis, total cellular RNA was isolated from the samples with Trizol (Invitrogen, Carlsbad, CA, USA) to evaluate the expression of chondrogenesis-related genes, including *Sox9*, *Col II*, and *Acan*, by real-time PCR (TaKaRa Bio, Tokyo, Japan). The reaction products were quantified using a relative quantification tool (CFX Manager, Bio-Rad) with *Gadph* as a reference gene. The primer sequences for *Acan*, *Sox9*, and *Col II* (Sango Biotech, Shanghai, China) are listed in Additional file 5: Table S2.

### Biomechanical assay for the elastic modulus of the regenerated articular cartilage

For the biomechanical assay of the elastic modulus of the tissue-engineered cartilage, we prepared condyles of each group into a cubic shape, being centered at the condylar cartilage defect and having smooth, parallel upper and lower surfaces. We measured and calculated the cross-sectional area and height of the sample before determining the longitudinal elastic modulus. The line of force of the loading device was set perpendicular to the sample surface before measurement. The ElectroForce® 3200 Series III test instrument (Bose Corporation Endura TEC Systems Group, Minnetonka, MN, USA) was applied for low-force testing of the engineered cartilage. The relative displacement and load were measured at a compression rate of 1 mm/min. The load–displacement curve was drawn with displacement as the abscissa axis and load as the vertical axis. In the initial stage of the load–displacement curve, we chose to analyze the relatively smooth, linear portion and calculated the elastic modulus (E) of the tissue-engineered cartilage: E = stress/strain, where stress = load/sectional area of the structure, and strain = relative displacement/height of the sample (see Additional file 10: Figure S8). Specimens of each group had three replicates at each time (2, 4, and 8 weeks).

### Statistical analysis

The results are expressed as the mean ± standard deviation and were compared by one-way analysis of variance (ANOVA) in combination with the Newman-Keuls post hoc test. The detection results were statistically analyzed using SPSS 17.0 (SPSS, USA). *P* < 0.05 was accepted as statistically significant.

## Results

### Microstructure and ultrastructure of the PRF, BMSC cell sheet, and BMSC/PRF construct

**Fig. 1:**
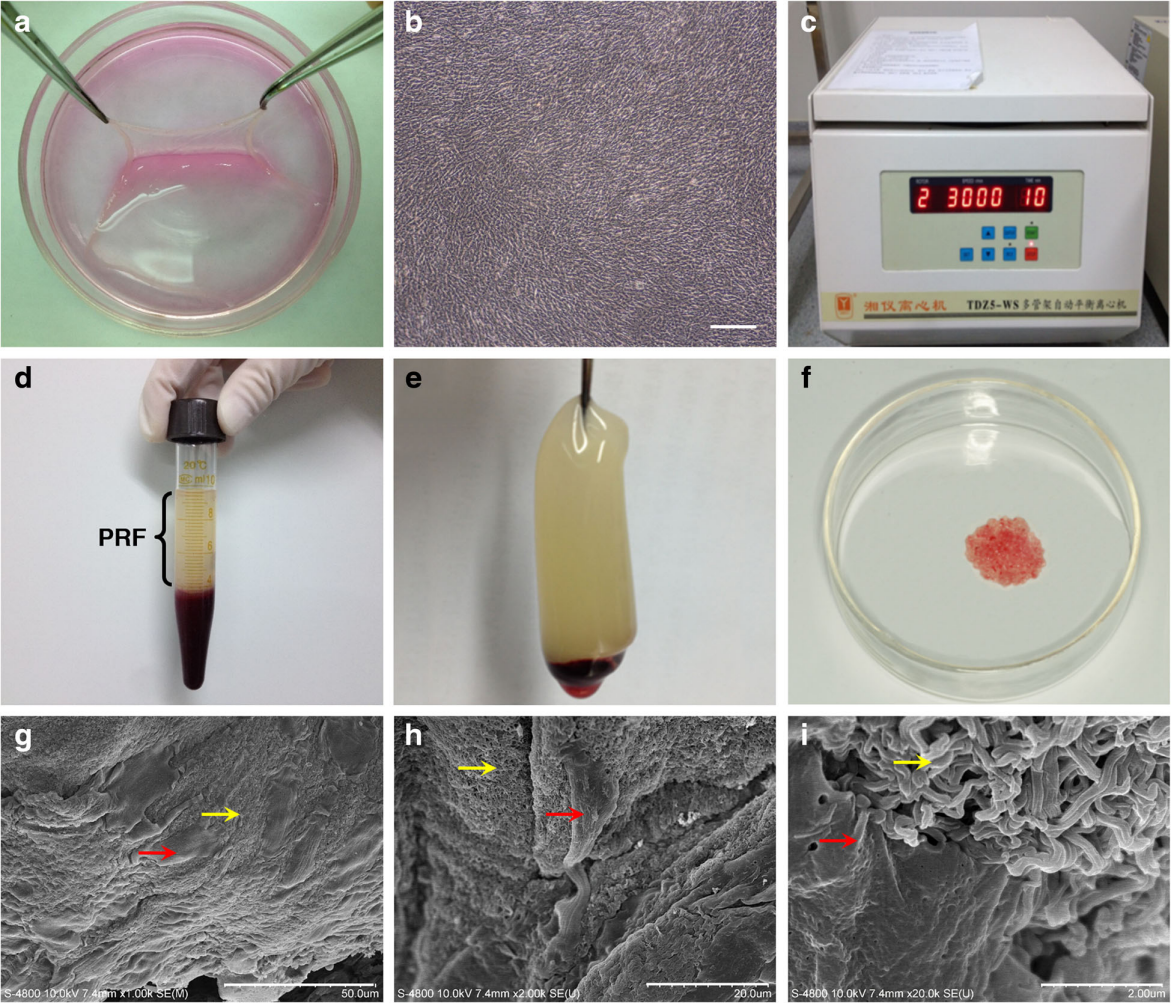
Structure of the platelet-rich fibrin (PRF). **a** A cell sheet that could be fully stripped was formed after 14 days of induction with cell sheet induction medium. **b** Inverted microscope observation showed the intertwined arrangement and stable growth morphology of the cell sheet (× 40, bar = 200 μm). **c** Blood samples (10 ml) were extracted from the central auricular arteries of rabbits and immediately centrifuged at 3000 r/min for 10 min. **d** PRF clot between the top layer of serum containing a few cells and the bottom layer of red corpuscles. **e** Complete removal of the PRF clot. **f** Preparation of the BMSC/PRF construct. **g–i** Cross-sectional observation by SEM. **g** Horizontal view at low magnification (× 1000). **h** Longitudinal view at low magnification (× 2000). **i** Longitudinal view at high magnification (× 20,000) (red arrow: BMSCs; yellow arrow: PRF)

Rabbit BMSCs were isolated and identified (see Additional file 1: Figure S1 and Additional file 2: Figure S2) and then were seeded in six-well plates in α-MEM containing 100 μg/ml vitamin C and 10% fetal bovine serum for 10–14 days. A milky sheet-like structure was formed at the bottom of all culture plates. The edge of the sheet was curled, and white granular nodules were found on the surface of the sheet. After being gently stripped off the plate, the sheet was slightly retracted with certain elasticity (Fig. 1a). Microscopic observation showed good growth of cells, which overlapped and appeared fusiform, in an intertwined arrangement (Fig. 1b). A 10-μl blood sample was rapidly extracted from the central auricular arteries of rabbits and centrifuged in glass tubes at 3000 r/min for 10 min (Fig. 1c). After centrifugation, a PRF clot was visible between the top layer of serum and bottom layer of red corpuscles (Fig. 1d). The glass tubes were allowed to stand for 10–15 min before the PRF clot was completely removed using tweezers (Fig. 1e). When the P3 generation of BMSCs formed the sheet, they were placed in sterile Petri dishes together with the prepared PRF granules. The two components were manually and thoroughly mixed at a particular ratio to obtain the rabbit BMSC/PRF construct (Fig. 1f). The ultra-structural features of the construct were examined from different angles using SEM after 4 days of mixed culture. In the cross section, we found many long, fusiform BMSCs adhering to the porous, reticular structure of PRF (Fig. 1g). In the longitudinal section, cells were embedded into the mesh-like structure of PRF by extending pseudopodia, which closely bound the two components (Fig. 1h, i). Owing to this mechanism, the BMSC/PRF construct formed a coherent whole, rather than two membranes stacked together.

### Determination of favorable hydrostatic pressure conditions for BMSC proliferation and chondrogenic differentiation in BMSC/PRF co-culture system

**Fig. 2:**
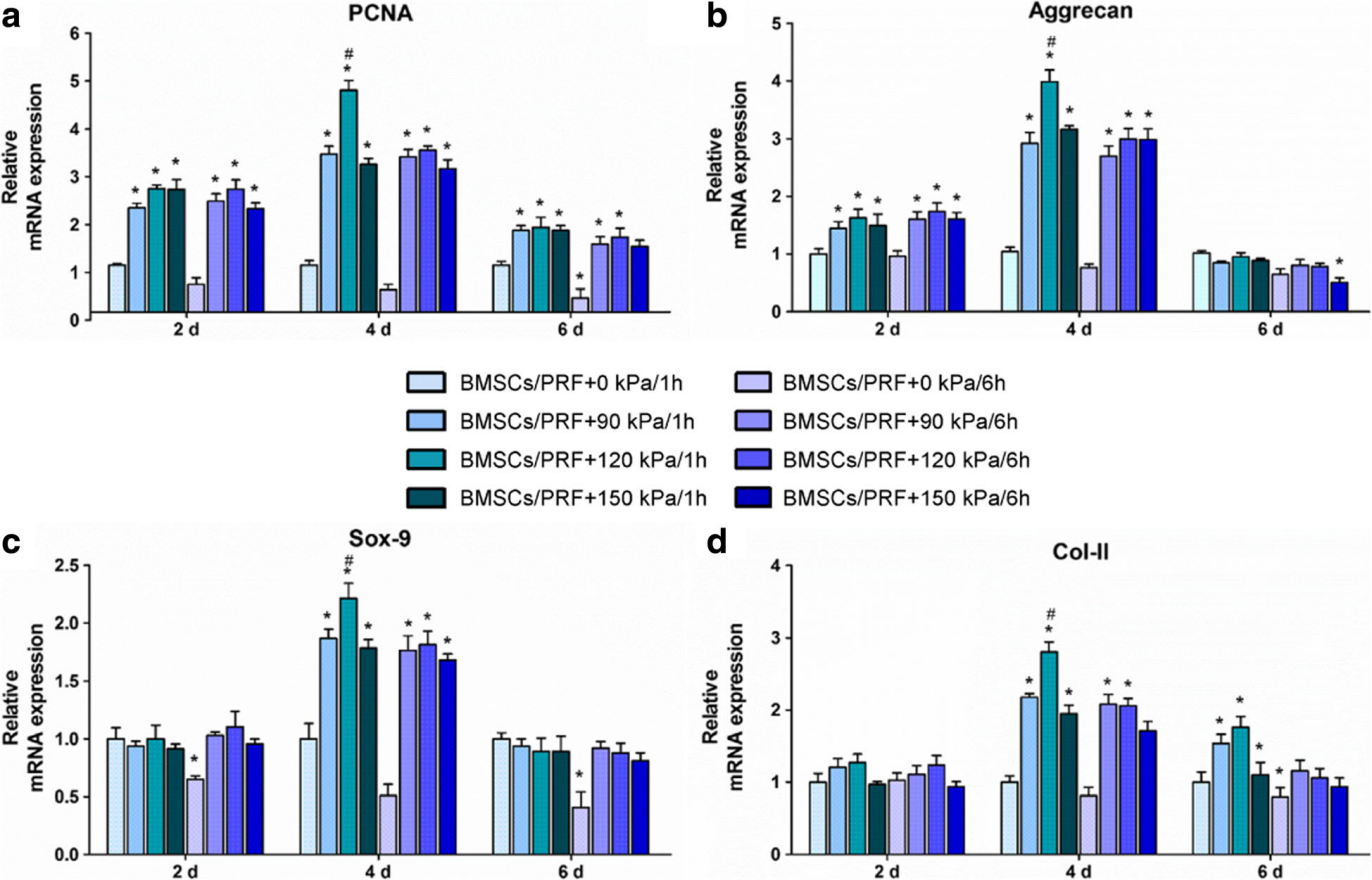
Real-time PCR assay of proliferation- and chondrogenesis-related gene expression in BMSCs of the BMSC/PRF construct under different conditions of mechanical stimulation. **a** *Pcna*. **b** *Acan*. **c** *Sox9*. **d** *Col II*. ^*^P < 0.05, vs. control group; ^#^*P* < 0.05, vs. indicated group

Real-time quantitative PCR analysis showed an overall trend of *proliferation* and *chondrogenic gene expression* in the BMSC sheets co-cultured with PRF and stimulated under different pressure conditions. The mRNA expression levels of cell proliferation-related *Pcna* (Fig. 2a) and chondrogenesis-related *Acan* (Fig. 2b), *Sox9* (Fig. 2c), and *Col II* (Fig. 2d) peaked in the cells at 4 days and began to decline at 6 days. Further analysis revealed that BMSCs of the construct showed higher *Pcan* and *Acan* mRNAs than the non-pressure group at 2 days (*P* < 0.001, *P* < 0.05), while no significant difference was found in *Sox9* and *Col II* between the pressure and non-pressure groups at the same time point (*P* > 0.05). BMSCs in the construct showed significantly higher *Pcna*, *Acan*, *Sox9*, and *Col II* levels than those in the control group at 4 days (*P* < 0.05). Under different pressure conditions, all four genes were expressed at the highest levels in the 120 kPa/1 h group, which showed significant differences compared with the other pressure groups at the same time points (*P* < 0.05). There was no significant difference between the pressure groups stimulated for 1 h and 6 h per day (*P* > 0.05). After 6 days of repeated pressure loading, only 90 and 120 kPa for 1 h per day exhibited a promoting effect on *Col II* mRNA. In contrast, a downward trend was found in the expression of proliferation-related genes and most chondrogenic differentiation-related genes in BMSCs under the pressure conditions with the three force values lasting for 6 h per day; however, the decreases were not statistically significant (*P* > 0.05). According to the quantitative PCR results in the different pressure conditions, we determined that 120 kPa, 1 h per day, for 4 days, was optimal for BMSC proliferation and chondrogenic differentiation.

### Effects of favorable hydrostatic pressure to the ultrastructure of the BMSCs

**Fig. 3:**
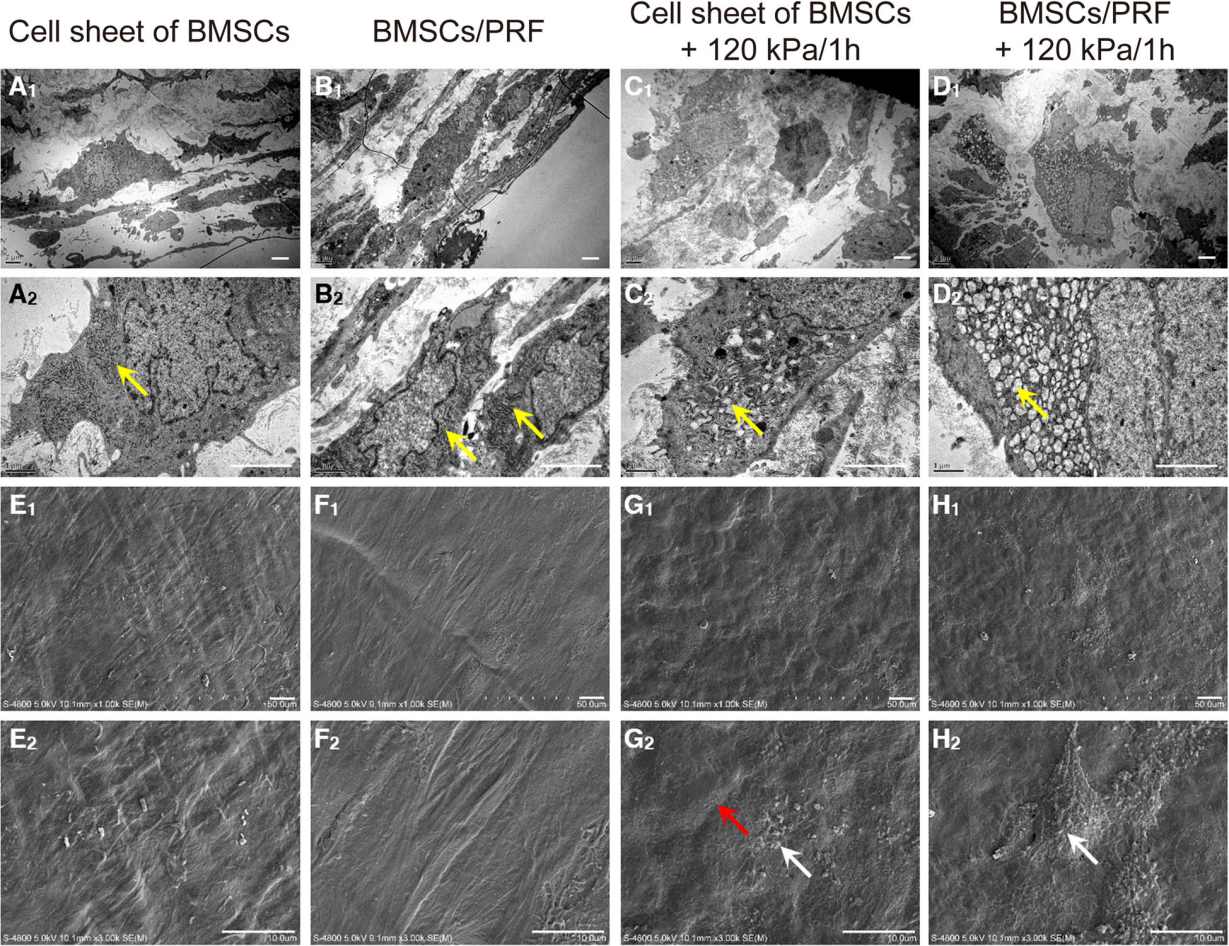
TEM and SEM images of the BMSC/PRF construct. **A**_**1**_**–D**_**1**_ TEM images at low magnification (× 5000, bar = 2 μm). **A**_**2**_**–D**_**2**_ TEM images at high magnification (× 10,000, bar = 1 μm,); yellow arrow points to the endoplasmic reticulum structure. **E**_**1**_**–H**_**1**_ SEM images at low magnification (× 1000, bar = 50 μm). **E**_**2**_**–H**_**2**_ SEM images at high magnification (× 3000, bar = 10 μm); white arrow points to granular secretions and red arrow points to reticular collagen fibers

TEM images showed a relatively closed state of endoplasmic reticulum in cells of the BMSC sheet (Fig. 3A_1, 2_) and that co-cultured with PRF (Fig. 3B_1, 2_). Partially dilated endoplasmic reticulum was found in BMSC sheets pre-pretreated with the abovementioned optimal mechanical stimulation (Fig. 3C_1, 2_), while the greatly dilated endoplasmic reticulum was observed in cells of the pressure-pretreated BMSC/PRF construct group (Fig. 3D_1, 2_). These results indicated that pressure stimulation at 120 kPa/1 h for 4 days enhanced the secretory function of BMSCs in the BMSC/PRF construct. SEM images revealed the presence of a large number of cell populations arranged densely on the surface of the cell sheet and wrapped by extracellular matrix (ECM) in the BMSC group (Fig. 3E_1,2_) and the BMSC/PRF group (Fig. 3F_1,2_). There was a small number of reticular collagen fibers and particulate proteoglycan-like secretions on the surface of the cell sheet in the pressure-pretreated BMSC sheet group, (Fig. 3G_1, 2_). In contrast, a large amount of reticular collagen fibers and particulate proteoglycan-like secretions was distributed on the surface of cell sheet in the pressure-pretreated BMSC/PRF construct group (Fig. 3H_1, 2_). These results indicated that optimal pressure stimulation promotes the secretion of cartilage matrix by BMSCs with PRF in existence.

### Cartilage regeneration after ectopic transplantation

**Fig. 4:**
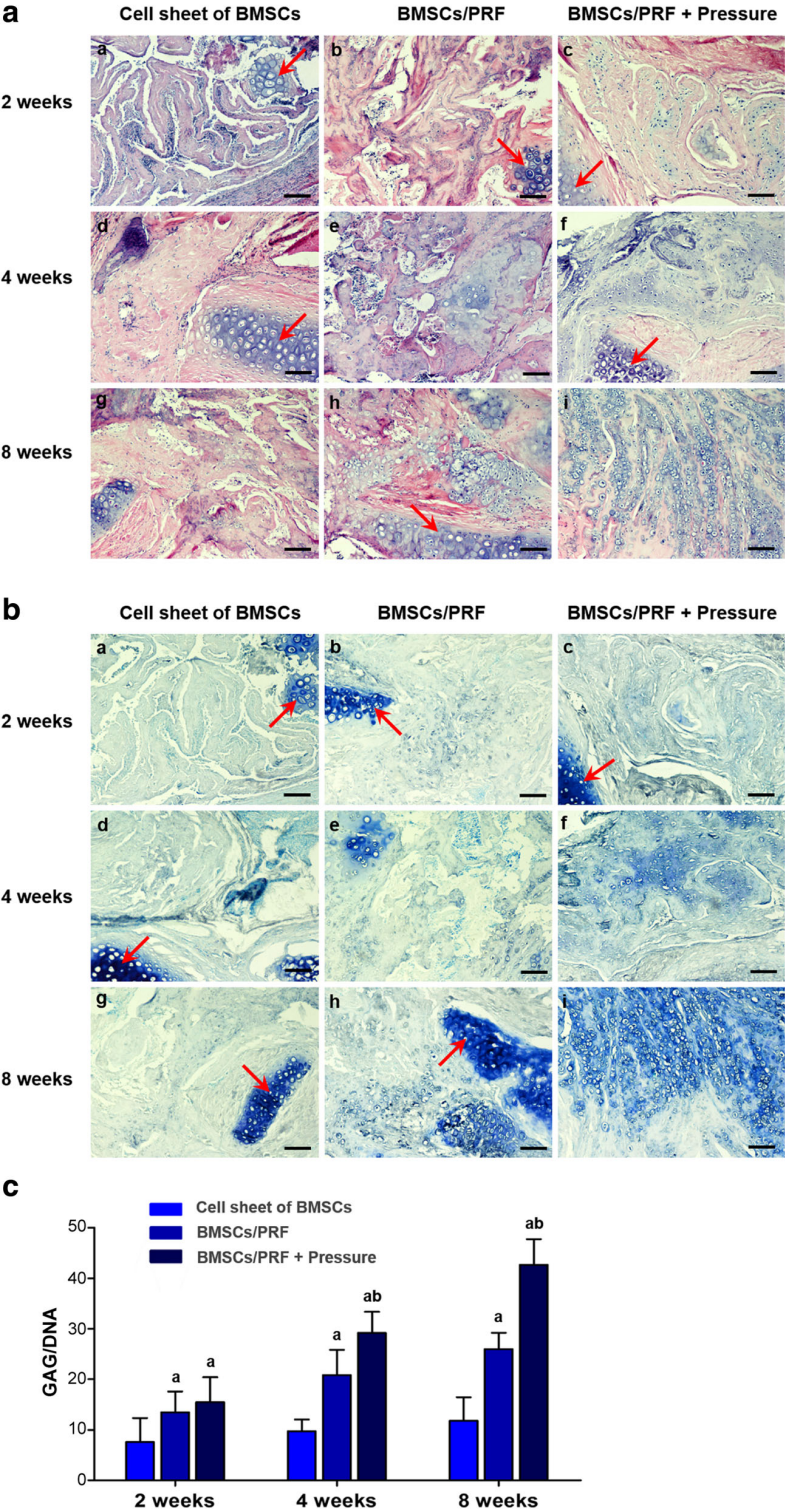
Histological and biomechanical examination of cartilage tissue regenerated in the subcutis of nude mice using the BMSC/PRF construct. **a** HE staining of specimens from nude mice. a–c BMSC sheet group at 2, 4, and 8 weeks, respectively; d–f BMSC/PRF construct group at 2, 4, and 8 weeks, respectively; and g–i pressure-pretreated BMSC/PRF construct group at 2, 4, and 8 weeks, respectively (red arrow indicates the implanted cartilage fragment; × 100, bar = 100 μm). **b** Toluidine blue staining of specimens from nude mice. a–c BMSC sheet group at 2, 4, and 8 weeks, respectively; d–f BMSC/PRF construct group at 2, 4, and 8 weeks, respectively; and g–i pressure-pretreated BMSC/PRF construct group at 2, 4, and 8 weeks, respectively (red arrow indicates the implanted cartilage fragment; × 100, bar = 100 μm). **c** Image analysis of toluidine blue staining intensity of specimens from nude mice. ^a^*P* < 0.05, vs. BMSC sheet transplantation group; ^b^*P* < 0.05, vs. no-pressure BMSC/PRF transplantation group

Forty-five nude mice had normal activities and eating after surgery. The wound healed well, and no swelling or oozing was observed. The transplant showed no significant absorption over time, and the surrounding tissue had no signs of necrosis or infection. Specimens were taken for staining and observation after 2, 4, or 8 weeks of in vivo culture in nude mice. HE staining showed that only a few cartilage-like cells were formed in the BMSC transplantation group at 8 weeks after transplantation. However, in the BMSC/PRF and pressure-pretreated BMSC/PRF transplantation groups, cartilage-like cells were distributed in the cell lacunae after 2, 4, and 8 weeks of culture in the subcutis of nude mice. The cartilage-like cells were surrounded by uniform ECM, and the number of cartilage-like cells formed gradually increased over time. However, the pressure-pretreated BMSC/PRF transplantation group formed a greater number of cartilage-like cells than the other groups at a given time point (Fig. 4A).

After TB staining, the neocartilage-like ECM was stained blue, similar to normal cartilage tissue (Fig. 4B). GAG/DNA ratio analysis revealed that the GAG content gradually increased over time in the BMSC/PRF and pressure-pretreated BMSC/PRF transplantation groups at 2, 4, and 8 weeks, with the GAG/DNA ratios significantly higher than that in the BMSC transplantation group at each given time point (*P* < 0.05). In particular, the pressure-pretreated BMSC/PRF transplantation group showed significantly higher GAG/DNA ratios than those of the no-pressure BMSC/PRF group at 4 and 8 weeks (*P* < 0.05) (Fig. 4C).

### General observation

**Fig. 5:**
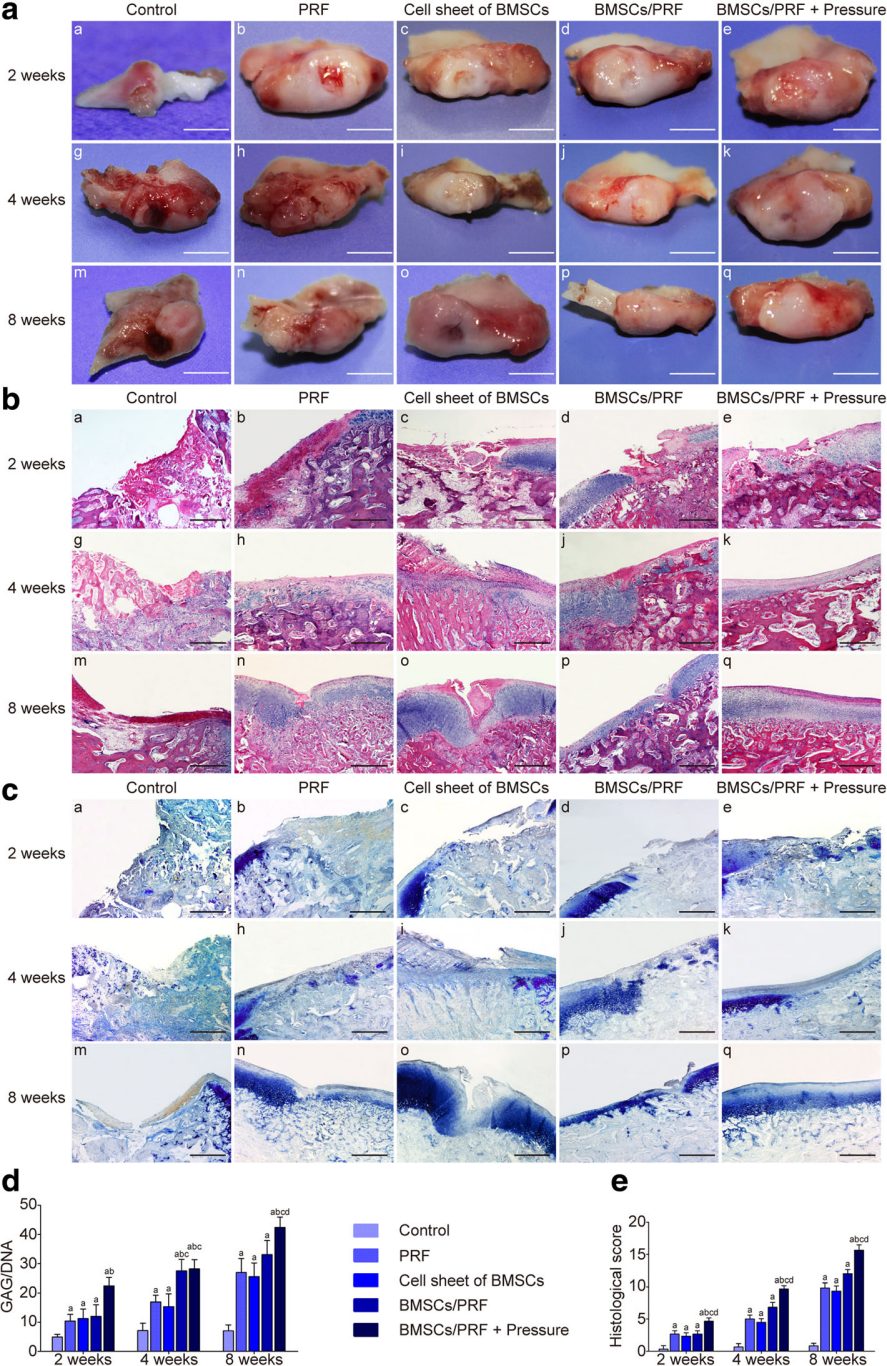
Histological and biomechanical examination of in situ regeneration and repair of condylar cartilage defects in rabbit using the BMSC/PRF construct. **a** Gross morphology of rabbit condyle (scale bar = 5 mm). **b** HE staining of rabbit condyle (× 40, bar = 250 μm). **c** Toluidine blue staining of rabbit condyle; a, g, and m control group at 2, 4, and 8 weeks, respectively; b, h, and n PRF group at 2, 4, and 8 weeks, respectively; c, i, and o BMSC group at 2, 4, and 8 weeks, respectively; d, j, and p BMSC/PRF group at 2, 4, and 8 weeks, respectively; e, k, and q pressure-pretreated BMSC/PRF construct group at 2, 4, and 8 weeks, respectively (× 40, bar = 250 μm). **d** Statistical analysis of GAG/DNA ratio of each specimen. **e** Morphological scores of repair tissue in rabbit condylar cartilage defects. ^a^*P* < 0.05, vs. control group; ^b^*P* < 0.05, vs. PRF transplantation group; ^c^*P* < 0.05, vs. BMSC sheet transplantation group; ^d^*P* < 0.05, vs. no-pressure BMSC/PRF transplantation group

Altogether, 176 New Zealand rabbits survived the surgery. The animals had normal activities and eating. No infection of the incision was observed. The growth condition of the animals was good (see Additional file 11: Movie S1 and Additional file 12: Movie S2). At 2, 4, and 8 weeks after surgery, the condylar articular surface of each group kept pyriform, greater in the front and smaller in the back. No periostosis or adhesion between condyle and articular disc was observed. Only in the blank control group, the tissue surrounding the defect site gradually absorbed over time, which made the cartilage defect clearly visible. In the other experimental groups, the defect was gradually filled with the repair tissue. Better results were always achieved in the pressure-pretreated BMSC/PRF transplantation group compared with other groups at the same time point. In the pressure-pretreated BMSC/PRF group, the repair tissue completely filled the defect at 12 weeks; there was no clear boundary from the surrounding cartilage, and the color and texture were generally consistent with those of normal cartilage (Fig. 5A).

### Histological staining observation

Hematoxylin and eosin (HE) staining showed that from 2 to 8 weeks after surgery, the defect site was depressed and only partially filled with fibrous tissue in the control group; cartilage cell differentiation was not observed in the repair tissue during the observation period, while absorption was obvious in the adjacent articular cartilage. In the individual PRF and BMSC groups, a cartilage-like substance was gradually formed in the defect over time; however, the arrangement of layers was disordered, and the proliferative, hypertrophic layers of adjacent cartilage showed significant thickening. In the BMSC/PRF group, the repair tissue in the defect site kept up with the general level to the adjacent normal tissue at 8 weeks. However, the repaired cartilage was relatively thin compared to the adjacent normal cartilage and there was a clear dividing line between them. In the pressure-pretreated BMSC/PRF group, better repair of the condylar defect was achieved compared with other groups at any given time point. The cartilage defect site was generally repaired at 8 weeks after surgery, with the cartilage surface being covered with dense fibrous tissue, which was thicker than the normal fibrous layer. The neo cartilage layers were clear and in good continuity with the layers of adjacent normal cartilage (Fig. 5B).

### Toluidine blue staining observation and GAG content evaluation

The neocartilage-like substance in the defects of each group was positive for TB staining, similar to normal cartilage tissue; however, the staining intensity varied between groups (Fig. 5C). Further analysis of the GAG content showed that the GAG/DNA ratio gradually increased in the four experimental groups (except the control group) from 2 to 4 to 8 weeks after surgery; the values were significantly higher than those of the control group at any given time point (*P* < 0.05). No significant difference was observed in the GAG/DNA ratio between the individual PRF and BMSC groups at each time point. However, the pressure-pretreated BMSC/PRF group showed significantly higher GAG/DNA ratio than the no-pressure BMSC/PRF group at the same time point (*P* < 0.05, Fig. 5D).

### Histomorphometric evaluation of regenerated cartilage

As we have defined before, animals were randomly assigned to five groups: blank control (natural recovery, group A), PRF alone (group B), BMSC sheet alone (group C), BMSC/PRF construct (group D), and pressure-pretreated BMSC/PRF construct (group E). Statistical analyses showed that groups B, C, D, and E had significantly higher scores than group A at any given time point (*P* < 0.05). In particular, group E had significantly higher scores than the other three groups at each time point (*P* < 0.05) (Fig. 5E).

### Real-time PCR assay for chondrogenic genes in regenerated cartilage

**Fig. 6:**
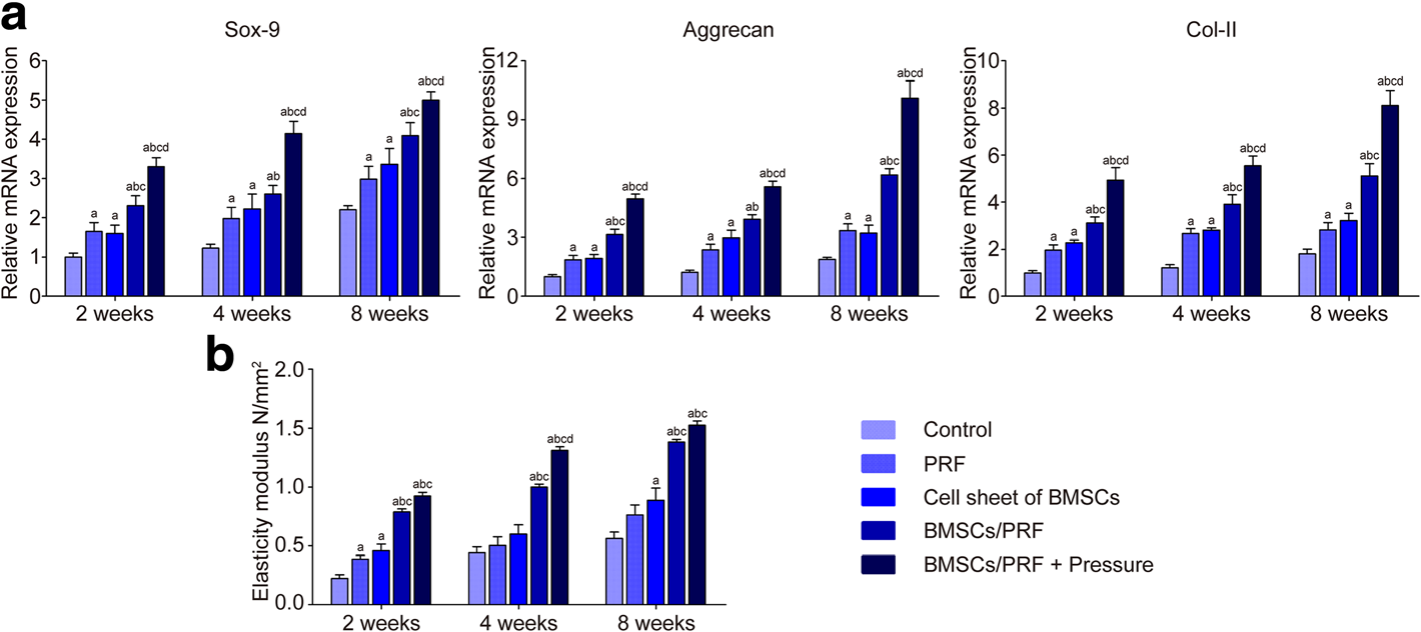
Real-time PCR assay of chondrogenesis-related gene expression in the repaired articular cartilage and biomechanical testing on the regenerated cartilage of each group. **a** Real-time PCR assay of chondrogenesis-related gene *Acan*, *Sox9*, and *Col II*. **b** Elastic modulus test in the repair region of rabbit condylar defects in different groups. ^a^*P* < 0.05, vs. control group; ^b^*P* < 0.05, vs. PRF transplantation group; ^c^*P* < 0.05, vs. BMSC sheet transplantation group; ^d^*P* < 0.05, vs. no-pressure BMSC/PRF transplantation group

The *ACAN* and *Sox9* expression levels in each group were significantly higher than those in the control group at any given time point, and they increased in a time-dependent manner (*P* < 0.05). Among the samples, the gene expression in the pressure-pretreated BMSC/PRF construct group was highest at each time point (*P* < 0.05) (Fig. 6a).

### Biomechanical testing on the regenerated cartilage

Biomechanical testing of neocartilage in the defect site showed that the elastic modulus of cartilage in the condylar center did not change significantly over time in the sham group. The elastic modulus in the other groups showed an upward trend over time and remained higher than the control group (*P* < 0.05). Data comparison between groups at various time points revealed that the condylar cartilage that regenerated in the BMSC/PRF transplantation group had significantly better biomechanical properties than the individual BMSCs or PRF transplantation group (*P* < 0.05). Among different experimental groups, the pressure-pretreated BMSC/PRF transplantation group obtained the most desired restoration of elastic modulus of cartilage, which was significantly higher than the other experimental groups (*P* < 0.05). The elastic modulus of cartilage in the pressure-pretreated BMSC/PRF transplantation group generally reached 75% of that in the positive control group at 8 weeks (Fig. 6b).

## Discussion

The temporomandibular joint (TMJ) is one of the most sophisticated and frequently used joints in humans. The mandibular condylar cartilage of the TMJ is a secondary and fibrous type of cartilage that is characterized by a different cell composition and hierarchical organization compared with the articular cartilage of the knee joint (hyaline cartilage) [26]. Biomechanical factors resulting from the functional activity of the mandibular joint are thought to influence the growth of the condyle, especially of the pressure-sensitive articular cartilage [27–30]. After articular cartilage injury in large joints, the mechanical stimulation during cartilage repair can be controlled through joint immobilization. However, in the case of TMJ, such loading control is impossible because eating is necessary for the animal, and mechanical stimulation therefore constantly exists during the articular cartilage repair process [31, 32]. For this reason, the mechanical adaptation of mandibular condylar cartilage may be more significant compared with the cartilage of larger joints (e.g., knee and hip joints) [33, 34]. From this perspective, the TMJ condylar cartilage can be regarded as an excellent model for studying the regeneration and repair of the mechanical adaptability and controllability of articular cartilage.

Many studies have tried to induce cartilage regeneration both in vitro and in vivo, but they are still far from obtaining regenerated cartilage that has properties similar to those of native cartilage, as well as integrating engineered and native tissue [35–37]. It was hypothesized that the lack of success was due to the lack of some critical factors and cues for chondrogenic cell differentiation and biomechanical adaption from the seed cells’ milieu [12, 38–40]. In the present study, we reveal, for what we believe is the first time, that feasible hydrostatic pressure effectively promotes the proliferation and chondrogenic differentiation of BMSCs in a BMSC/PRF construct, and the transplantation of a mechanically pretreated construct made from BMSC sheet fragments and PRF granules could obviously improve the regeneration and repair quality of articular cartilage defects, especially the integration between the regenerated cartilage and host cartilage milieu. Our findings provide us with a new tissue-engineering cartilage transplant with degradable grafts, good biocompatibility, sustained release of multiple growth factors and biomechanical regulation potential. The newly formed construct with biomechanical flexibility showed a superior capacity for cartilage regeneration by promoting the boundaryless repair of full-thickness defects of articular cartilage. It can not only provide new ideas for the treatment of condylar cartilage defects but can also provide valuable research experience for the microenvironmental optimization of tissue-engineering treatments for various articular cartilage defects in other general joints.

We noticed that both the proliferation and chondrogenic markers of the BMSC/PRF construct decreased upon treatment under 120 kPa/1 h for 6 days compared with that for 4 days. One possible reason might be that the relevant mechanotransduction signal molecules in the cells are fully activated under pressure stimulation for 4 days, thereby regulating the expression of the BMSC cell proliferation genes and cartilage-related genes to the greatest extent. However, under 120 kPa/1 h for 6 days, as the sensitivity of the cells to mechanical stimuli might possibly decrease and the concentration of sustained-release growth factors in the PRF also decreased, the vigorous growth and secretion state of the cells can no longer be maintained at the highest level. To test our speculation, ELISA was performed to detect the release of four important growth factors (TGF-β, IGF-1, VEGF, and EGF) by the PRF membrane in the BMSC/PRF construct for 4 days. Studies have found that TGF-β1 [41–46], PDGF [47–49], IGF-1 [44–46, 50–52], and EGF [53] all have the function of promoting the differentiation of stem cells into chondrocytes. The results showed that the release of TGF-β, VEGF, and EGF was highest at day 1 and then gradually decreased on days 2–4. The release of IGF-1 peaked at day 2 and then gradually decreased afterwards (Table S1). Therefore, we speculate that the decrease in both the proliferation and chondrogenic markers in the BMSC/PRF construct under 120 kPa/1 h for 6 days might be related to the decreased growth factor release from the PRF or even the decreased synergistic effects between the mechanical stimulation and growth factors, which still needs further study.

In both ectopic and in situ cartilage regeneration experiments in the present work, we found that a large number of neocartilage-like tissues formed even in the PRF-free BMSC transplantation group, but the quality and quantity of the neocartilage were inferior to those in the BMSC/PRF group. This showed from another point of view that BMSCs have limited chondrogenic differentiation potential without the support of PRF. Additionally, the use of local high concentrations of growth factors in a cartilage defect in situ can promote cartilage defect repair through cell homing [54]. In an in situ rabbit condylar cartilage regeneration experiment, we also found that a large number of cartilage-like tissues filled the defect at 8 weeks after the transplantation of PRF alone. These data suggest that, to a certain degree, PRF can activate the recruitment of intrinsic stem/progenitor cells to the damaged site and further support the lasting chondrogenesis of the PRF. According to the above analysis, we concluded that PRF can promote cartilage regeneration by simultaneously facilitating the homing and chondrogenic differentiation of exogenous seed cells in the repair of oversized defects in the mandibular condylar cartilage.

In traditional cartilage tissue engineering, seed cells cultured in vitro lack mechanical stimuli, resulting in cartilage tissues with the disadvantages of irregular cell arrangement and low cartilage matrix content [33]. The inevitable load-bearing property of tissue-engineered articular cartilage used for cartilage defect repair in vivo makes it easier to achieve the continuous mechanical stimulation of seed cells than by using sustained growth factor stimulation. To reveal the effect of the mechanical milieu on cells or tissues in vivo, many studies have performed in vitro cell mechanics experiments and designed particular loading methods (e.g., mechanical type, size, time, and frequency) to observe the effect of mechanical stimulation on cells [55]. Because stem cells have increased mechanical sensitivity compared to adult cells [13], biomechanical signals may play key roles in the regulation of the phenotypic differentiation of stem cells [14]. They can sense the mechanical properties and perceive mechanical information that directs broad aspects of cell functions, including lineage commitment. However, although mechanical stimulation may be able to enhance the regeneration process of articular cartilage [56], the underlying molecular regulation mechanism is far from clear. Several studies have pointed out that the Ihh/PTHrP signaling pathways in condylar cartilage are related to stress [50, 57]. In addition, it has been reported that TGF-β/Smad is involved in the mechanically stimulated development and repair of condylar artilage [58, 59]. We also carried out a series of studies on BMSCs’ mechanotransduction mechanism, which confirmed that P38MAPK/NF-κB signaling, Wnt signaling, and BMP/Smads signaling all participate in the mechanotransduction process of BMSCs. To better understand the mechanical and biological responses and the signal transduction mechanisms of BMSCs in response to stress, stable isotope labeling by amino acid (SILAC) detection in BMSCs was further used to screen differentially expressed signal molecules after mechanical stimulation. We found that a novel mechanosensor, anthrax toxin protein receptor 1 (ANTXR1), plays a crucial role in BMSC mechanotransduction (unpublished data, Additional files 1, 2, 3, 4, 5 and 6: Figures S1, S2, S3, S4; Tables S1, S2).

Previous studies have found that the proliferation and differentiation abilities of BMSCs changed under certain mechanical stimulations [60–67]. In the present study, we further found that, in the mechanically pretreated BMSC/PRF construct transplant group, the repaired tissue had no clear boundary with the surrounding normal cartilage after 8 weeks of repair. Such superior regeneration may be associated with our improvements of the tissue-engineered cartilage in the following three aspects. First, a particular hydrostatic pressure condition can significantly stimulate the gene expression of the proliferation-related *Pcna* and chondrogenesis-related *Acan*, *Sox9*, and *Col-II* in BMSCs. Moreover, we found through in situ regeneration and repair experiments of condylar cartilage defects that cartilage-like cells were poorly arranged in the generated neocartilage after the transplantation of BMSCs without mechanical stimulation; the hierarchical arrangement of the cells was far less ordered than those in the experimental group transplanted with BMSCs pretreated by mechanical stimulation. This suggests that the mechanical adjustment of the seed cells may also have a great impact on the polar arrangement and secretion direction of the cells. Second, PRF, as a new type of cartilage scaffold and multiple growth factor carrier, enables the tissue-engineered cartilage constructed from BMSC sheet fragments and PRF granules to directly bond to the adjacent cartilage. Third, the elastic modulus of the articular cartilage 8 weeks after repair using the pressure-pretreated BMSC/PRF construct exceeded 70% of that in normal cartilage. Thus, the differences in composition and biomechanics between the neocartilage and surrounding host cartilage could be effectively narrowed, and the cartilage-to-cartilage integration and overall biomechanical properties of the repaired cartilage could be improved. In another work, we will further explore the mechanical signal transduction mechanism of adult stem cells in response to mechanical stimuli and the synergistic effect of mechanical stimulation and growth factors, which will contribute to the ideal functional regeneration and repair of articular cartilage defects.

## Conclusion

Many studies have tried to induce articular cartilage regeneration both in vitro and in vivo; however, we are still far from obtaining regenerated articular cartilage that has the properties similar to the native cartilage, as well as integrating engineered and native tissue. Our study provides a novel method of transplant construction with PRF as a source of multiple growth factors and an autologous scaffold for tissue-engineered articular cartilage repair. We obtained the first evidence that appropriate pressure pretreatment can maximize the chondrogenic differentiation potential of seed cells in the novel BMSC/PRF tissue-engineered cartilage transplant. We found that implanting the pressure-pretreated construct can effectively improve the regeneration and repair rate, matrix content, and mechanical parameters of neocartilage, thereby achieving boundaryless repair between the neocartilage and residual host cartilage and ultimately realizing integration with the host cartilage milieu and functional regeneration and repair. Our work demonstrates that pressure is an indispensable factor that promotes tissue-engineered regeneration and repair of TMJ articular cartilage and plays a crucial role in improving the mechanical properties and integration of neocartilage.

## Additional files

Additional file 1: **Figure S1.** Isolation and identification of BMSCs. (A) Cells grew rapidly, and small colonies gradually merged, with radial growth in primary culture at 7–9 days (× 40, bar = 200 μm). (B) The third generation of cells were long, fusiform, and densely arranged, in a swirl pattern (× 40, bar = 200 μm). (C-F) Flow cytometry indicated that the BMSCs were negative for hematopoietic markers CD34 and CD45 but positive for mesenchyme-associated markers CD29 and CD44. Representative figures showing the multi-directional differentiation of PDLSCs. (G) Mineralized nodules were formed after 4 weeks of osteogenic induction (stained with alizarin red, (× 40, bar = 200 μm). (I) Alkaline phosphatase staining showed a large number of blue metachromatic regions. (K) Lipid vacuoles were observed after 2 weeks of adipogenic induction (stained with oil red O, (× 40, bar = 200 μm). (H, J and L) Uninduced control cells were negative for alizarin red, alkaline phosphatase, and oil red O staining. (TIF 14270 kb)

Additional file 2: **Figure S2.** Growth curve of BMSCs in the BMSC/PRF construct (*P < 0.05, vs. control group). (TIF 422 kb)

Additional file 3: **Table S1.** Detection of continued growth factor release from PRF in the BMSC/PRF construct over 96 h. (DOCX 14 kb)

Additional file 4: **Figure S3.** The multi-functional pressure loading system for in vitro cultured cells. (A) Overall view of the system. (B) Control panel. (C) Drive control system–compression pump. (D) Drive control system–vacuum pump. (E) Lateral view of the pressure incubator. (F) Front view of the pressure incubator. (G) Interior of the pressure incubator. (H) Schematic illustration of the whole system. (TIF 8531 kb)

Additional file 5: **Table S2.** Primers used in Real-time PCR. (DOCX 13 kb)

Additional file 6: **Figure S4.** Construction of the stem cell/PRF construct (inverted phase contrast microscopy) Group I: The PRF membrane was partially suspended. Only the thinner edge was slightly adhered to the bottom of the plate, while the thicker portion was suspended. No obvious cell proliferation was observed around the PRF membrane. Many red corpuscles were suspended in the culture medium **(Figure S4A).** Group II: Under the microscope, the cell sheet wrapping the PRF fragments formed an opaque construct **(Figure S4B).** Group III: Under the microscope, the cell sheet wrapping the PRF fragments formed an opaque construct as Group II. Group IV: The PRF granules and cell sheet fragments were mechanically embedded into each other and integrally suspended in the culture medium **(Figure S4C).** A small number of cell sheet fragments were scattered at the bottom of the culture plate. The latter could re-colonize at the bottom of the plate, and there were cells migrating from its edges, showing radial proliferation **(Figure S4D).** Group V: The PRF membrane did not adhere well to the bottom of the plate. No aggregative cell growth was observed at the edge of the PRF membrane. The PRF membrane was shaking with the culture broth **(Figure S4E).**;Group VI: Most PRF granules were colonized at the bottom of the Petri dish. Numerous cells showed intensive radial growth in interleaved multi-layers around the PRF granules. The cell density was higher than that in the peripheral PRF-free region **(Figure S4F).** (TIF 4296 kb)

Additional file 7: **Figure S5.** SEM images of the construct in each group. (A1, A2) Group I. (B1, B2, C1, C2) Groups II and III. (D1, D2) Group IV. (E1, E2) Group V. (F1, F2) Group VI. Group I: The PRF structure remained in most areas of the sample. Cell structure was only observed at the thinner edge of the PRF adhered to the bottom of the plate. **(Figure S5A1).** Only small amount of cells became flat and evenly laid on the PRF surface; however, there was less ECM on the cell surface **(Figure S5A2).** Groups II and III: The cell sheet was observed retaining an intact structure when it was co-cultured with the PRF by wrapping it **(Figure S5B1).** In the cross-section, only the three-dimensional reticular, crosslinked structure of the PRF was observed, in the absence of any cell structure **(Figure S5B2).** In a few cross-sections, aggregation of red and white corpuscles **(Figure S5C1)** and platelets **(Figure S5C2)** was observed in the red end of the PRF, namely the fibrous scaffold. Group IV: In the cell sheet fragment/PRF granule co-culture group, the cell sheet was mostly curled, and a large amount of ECM (white arrow) was present on the cell surface. In the cell sheet, cells extended numerous synapses to the surface and reticular structure voids of the PRF **(Figure S5D1, 2).** Group V: The PRF surface was completely covered by a sheet-like cell layer after the two inoculations **(Figure S5E1).** No extension of cell structure was observed into the reticular voids **(Figure S5E2).** Group VI: In this group, the entire surface of the granular PRF was almost covered by the sheet-like structure of cells **(Figure S5F1).** Cell pseudopodia extended from the surface of different PRF granules and connected to the surface of other PRF granules (**Figure S5F2**). (TIF 10744 kb)

Additional file 8: **Figure S6.** Establishment of a cartilage defect model in bilateral temporomandibular joints. (A) An approximately 2-cm-long skin incision was made from 5 mm outside the outer canthus to the external auditory canal. (B) The joint capsule was cut open. (C) The condylar articular surface was exposed. (D, E) an approximately 3-mm-diameter hole was drilled in the center of the anterior condyle incline. (F) The joint capsule was sutured. (TIF 4677 kb)

Additional file 9: **Table S3.** Criteria for semi-quantitative histological scoring of articular cartilage. (DOCX 14 kb)

Additional file 10: **Figure S8.** The test instrument was applied for low-force testing of the mandibular condylar cartilage. For the biomechanical assay of the elastic modulus of the tissue-engineered cartilage, we prepared each group of condyles into a cubic shape with smooth and parallel upper and lower surfaces **(Figure S8A, B).** The ElectroForce® 3200 Series III test instrument (Bose Corporation Endura TEC Systems Group, Minnetonka, MN, USA) was applied for low-force testing of the engineered cartilage. We measured and calculated the cross-sectional area and height of the sample before determining the longitudinal elastic modulus. The line of force of the loading device was set perpendicular to the sample surface before measurement **(Figure S8C, D).** The ElectroForce® 3200 Series III test instrument (Bose Corporation Endura TEC Systems Group, Minnetonka, MN, USA) was applied for low-force testing of the engineered cartilage. The relative displacement and load were measured at a compression rate of 1 mm/min. The load–displacement curve was drawn with displacement as the abscissa axis and load as the vertical axis **(Figure S8E).** The elastic modulus (E) of the neocartilage was calculated according to the measured load–displacement curve **(Figure S8F).** (TIF 1780 kb)

Additional file 11: **Movie S1.** Video of experimental animals chewing food before surgery. (MP4 16591 kb)

Additional file 12: **Movie S2**. Video of experimental animals chewing food 7 days after surgery. (MP4 34285 kb)

## Data Availability

The datasets generated during and/or analyzed during the current study are available from the corresponding author on reasonable request.

## Abbreviations

α-MEM: Minimum Essential Medium-α
Acan: Aggrecan
ANOVA: Analysis of variance
BMSCs: Bone marrow mesenchymal stem cells
Col: Collagen
ECM: Extracellular matrix
GAG: Glycosaminoglycans
HE: Hematoxylin and eosin
Pcna: Proliferating cell nuclear antigen
PCR: Polymerase chain reaction
PRF: Platelet-rich fibrin
SEM: Scanning electron microscopy
Sox9: SRY-related HMG box-9
TB: Toluidine blue
TEM: Transmission electron microscopy
TMJ: Temporomandibular joint

## References

[1] Iwamoto M, Ohta Y, Larmour C, Enomoto-Iwamoto M (2013). Toward regeneration of articular cartilage. Birth Defects Res C Embryo Today.

[2] Elder BD, Athanasiou KA (2009). Hydrostatic pressure in articular cartilage tissue engineering: from chondrocytes to tissue regeneration. Tissue Eng Part B Rev.

[3] Oldershaw RA (2012). Cell sources for the regeneration of articular cartilage: the past, the horizon and the future. Int J Exp Pathol.

[4] Tuan RS, Chen AF, Klatt BA (2013). Cartilage regeneration. J Am Acad Orthop Surg.

[5] Fridenshtein A, Piatetskii S, Petrakova KV (1969). Osteogenesis in transplants of bone marrow cells. Arkh Anat Gistol Embriol.

[6] Wakitani S, Goto T, Pineda SJ, Young RG, Mansour JM, Caplan AI (1994). Mesenchymal cell-based repair of large, full-thickness defects of articular cartilage. J Bone Joint Surg Am.

[7] Pastides P, Chimutengwende-Gordon M, Maffulli N, Khan W (2013). Stem cell therapy for human cartilage defects: a systematic review. Osteoarthr Cartil.

[8] Niemeyer P, Pestka JM, Kreuz PC, Erggelet C, Schmal H, Suedkamp NP (2008). Characteristic complications after autologous chondrocyte implantation for cartilage defects of the knee joint. Am J Sports Med.

[9] Huey DJ, Hu JC, Athanasiou KA (2012). Unlike bone, cartilage regeneration remains elusive. Science..

[10] Hollander AP, Dickinson SC, Kafienah W (2010). Stem cells and cartilage development: complexities of a simple tissue. Stem Cells.

[11] Ochi M, Uchio Y, Tobita M, Kuriwaka M (2001). Current concepts in tissue engineering technique for repair of cartilage defect. Artif Organs.

[12] Tenney RM, Discher DE (2009). Stem cells, microenvironment mechanics, and growth factor activation. Curr Opin Cell Biol.

[13] Chowdhury F, Na S, Li D, Poh YC, Tanaka TS, Wang F (2010). Material properties of the cell dictate stress-induced spreading and differentiation in embryonic stem cells. Nat Mater.

[14] Castillo AB, Jacobs CR (2010). Mesenchymal stem cell mechanobiology. Curr Osteoporos Rep.

[15] Kobayashi N, Yasu T, Ueba H, Sata M, Hashimoto S, Kuroki M (2004). Mechanical stress promotes the expression of smooth muscle-like properties in marrow stromal cells. Exp Hematol.

[16] Koike M, Shimokawa H, Kanno Z, Ohya K, Soma K (2005). Effects of mechanical strain on proliferation and differentiation of bone marrow stromal cell line ST2. J Bone Miner Metab.

[17] Thorpe SD, Buckley CT, Vinardell T, O'Brien FJ, Campbell VA, Kelly DJ (2008). Dynamic compression can inhibit chondrogenesis of mesenchymal stem cells. Biochem Biophys Res Commun.

[18] Chen YJ, Zhang M, Wang JJ (2007). Study on the effects of mechanical pressure to the ultrastructure and secretion ability of mandibular condylar chondrocytes. Arch Oral Biol.

[19] Zhang M, Wang JJ, Chen YJ (2006). Effects of mechanical pressure on intracellular calcium release channel and cytoskeletal structure in rabbit mandibular condylar chondrocytes. Life Sci.

[20] Zhang M, Chen YJ, Ono T, Wang JJ (2008). Crosstalk between integrin and G protein pathways involved in mechanotransduction in mandibular condylar chondrocytes under pressure. Arch Biochem Biophys.

[21] Zhang M, Chen FM, Wang AH, Chen YJ, Lv X, Wu S (2012). Estrogen and its receptor enhance mechanobiological effects in compressed bone mesenchymal stem cells. Cells Tissues Organs.

[22] Zhao YH, Zhang M, Liu NX, Lv X, Zhang J, Chen FM (2013). The combined use of cell sheet fragments of periodontal ligament stem cells and platelet-rich fibrin granules for avulsed tooth reimplantation. Biomaterials..

[23] Loken S, Jakobsen RB, Aroen A, Heir S, Shahdadfar A, Brinchmann JE (2008). Bone marrow mesenchymal stem cells in a hyaluronan scaffold for treatment of an osteochondral defect in a rabbit model. Knee Surg Sports Traumatol Arthrosc.

[24] Park K, Huang J, Azar F, Jin RL, Min BH, Han DK (2006). Scaffold-free, engineered porcine cartilage construct for cartilage defect repair--in vitro and in vivo study. Artif Organs.

[25] Ramallal M, Maneiro E, Lopez E, Fuentes-Boquete I, Lopez-Armada MJ, Fernandez-Sueiro JL (2004). Xeno-implantation of pig chondrocytes into rabbit to treat localized articular cartilage defects: an animal model. Wound Repair Regen.

[26] Zhang M, Chen FM, Chen YJ, Wu S, Lv X, Zhao RN (2011). Effect of mechanical pressure on the thickness and collagen synthesis of mandibular cartilage and the contributions of G proteins. Mol Cell Biomech.

[27] Aliko A, Ciancaglini R, Alushi A, Tafaj A, Ruci D (2011). Temporomandibular joint involvement in rheumatoid arthritis, systemic lupus erythematosus and systemic sclerosis. Int J Oral Maxillofac Surg.

[28] Bessa-Nogueira RV, Vasconcelos BC, Duarte AP, Goes PS, Bezerra TP (2008). Targeted assessment of the temporomandibular joint in patients with rheumatoid arthritis. J Oral Maxillofac Surg.

[29] Kuroda S, Tanimoto K, Izawa T, Fujihara S, Koolstra JH, Tanaka E (2009). Biomechanical and biochemical characteristics of the mandibular condylar cartilage. Osteoarthr Cartil.

[30] Wu M, Xu T, Zhou Y, Lu H, Gu Z (2013). Pressure and inflammatory stimulation induced increase of cadherin-11 is mediated by PI3K/Akt pathway in synovial fibroblasts from temporomandibular joint. Osteoarthr Cartil.

[31] Mio K, Kirkham J, Bonass WA (2007). Possible role of extracellular signal-regulated kinase pathway in regulation of Sox9 mRNA expression in chondrocytes under hydrostatic pressure. J Biosci Bioeng.

[32] Fanning PJ, Emkey G, Smith RJ, Grodzinsky AJ, Szasz N, Trippel SB (2003). Mechanical regulation of mitogen-activated protein kinase signaling in articular cartilage. J Biol Chem.

[33] Athanasiou KA, Responte DJ, Brown WE, Hu JC (2015). Harnessing biomechanics to develop cartilage regeneration strategies. J Biomech Eng.

[34] Brady MA, Sivananthan S, Mudera V, Liu Q, Wiltfang J, Warnke PH (2011). The primordium of a biological joint replacement: coupling of two stem cell pathways in biphasic ultrarapid compressed gel niches. J Craniomaxillofac Surg.

[35] Medvedeva EV, Grebenik EA, Gornostaeva SN, Telpuhov VI, Lychagin AV, Timashev PS (2018). Repair of damaged articular cartilage: current approaches and future directions. Int J Mol Sci.

[36] Duarte Campos DF, Drescher W, Rath B, Tingart M, Fischer H (2012). Supporting biomaterials for articular cartilage repair. Cartilage..

[37] Kwon H, Paschos NK, Hu JC, Athanasiou K (2016). Articular cartilage tissue engineering: the role of signaling molecules. Cell Mol Life Sci.

[38] Huang BJ, Hu JC, Athanasiou KA (2016). Cell-based tissue engineering strategies used in the clinical repair of articular cartilage. Biomaterials..

[39] Lundborg G (1999). The bone and joint decade 2000-2010. Scand J Plast Reconstr Surg Hand Surg.

[40] Getgood A, Brooks R, Fortier L, Rushton N (2009). Articular cartilage tissue engineering: today's research, tomorrow’s practice?. J Bone Joint Surg Br.

[41] Arora A, Mahajan A, Katti DS (2017). TGF-beta1 presenting enzymatically cross-linked injectable hydrogels for improved chondrogenesis. Colloids Surf B Biointerfaces.

[42] Xia P, Wang X, Qu Y, Lin Q, Cheng K, Gao M (2017). TGF-beta1-induced chondrogenesis of bone marrow mesenchymal stem cells is promoted by low-intensity pulsed ultrasound through the integrin-mTOR signaling pathway. Stem Cell Res Ther.

[43] Asen AK, Goebel L, Rey-Rico A, Sohier J, Zurakowski D, Cucchiarini M (2018). Sustained spatiotemporal release of TGF-beta1 confers enhanced very early chondrogenic differentiation during osteochondral repair in specific topographic patterns. FASEB J.

[44] Gugjoo MB, Amarpal, Abdelbaset-Ismail A, Aithal HP, Kinjavdekar P, Pawde AM (2017). Mesenchymal stem cells with IGF-1 and TGF- beta1 in laminin gel for osteochondral defects in rabbits. Biomed Pharmacother.

[45] Morscheid S, Rey-Rico A, Schmitt G, Madry H, Cucchiarini M, Venkatesan JK (2019). Therapeutic Effects of rAAV-Mediated Concomittant Gene Transfer and Overexpression of TGF-beta and IGF-I on the Chondrogenesis of Human Bone-Marrow-Derived Mesenchymal Stem Cells. Int J Mol Sci.

[46] Fukumoto T, Sperling JW, Sanyal A, Fitzsimmons JS, Reinholz GG, Conover CA (2003). Combined effects of insulin-like growth factor-1 and transforming growth factor-beta1 on periosteal mesenchymal cells during chondrogenesis in vitro. Osteoarthr Cartil.

[47] Yao Z, Chen P, Wang S, Deng G, Hu Y, Lin Q (2019). Reduced PDGF-AA in subchondral bone leads to articular cartilage degeneration after strenuous running. J Cell Physiol.

[48] Ataliotis P (2000). Platelet-derived growth factor a modulates limb chondrogenesis both in vivo and in vitro. Mech Dev.

[49] Lohmann CH, Schwartz Z, Niederauer GG, Carnes DL, Dean DD, Boyan BD (2000). Pretreatment with platelet derived growth factor-BB modulates the ability of costochondral resting zone chondrocytes incorporated into PLA/PGA scaffolds to form new cartilage in vivo. Biomaterials..

[50] Zhou Q, Li B, Zhao J, Pan W, Xu J, Chen S (2016). IGF-I induces adipose derived mesenchymal cell chondrogenic differentiation in vitro and enhances chondrogenesis in vivo. In Vitro Cell Dev Biol Anim.

[51] Ikeda Y, Sakaue M, Chijimatsu R, Hart DA, Otsubo H, Shimomura K (2017). IGF-1 gene transfer to human synovial MSCs promotes their chondrogenic differentiation potential without induction of the hypertrophic phenotype. Stem Cells Int.

[52] Uebersax L, Merkle HP, Meinel L (2008). Insulin-like growth factor I releasing silk fibroin scaffolds induce chondrogenic differentiation of human mesenchymal stem cells. J Control Release.

[53] Jia H, Ma X, Tong W, Doyran B, Sun Z, Wang L (2016). EGFR signaling is critical for maintaining the superficial layer of articular cartilage and preventing osteoarthritis initiation. Proc Natl Acad Sci U S A.

[54] Lee CH, Cook JL, Mendelson A, Moioli EK, Yao H, Mao JJ (2010). Regeneration of the articular surface of the rabbit synovial joint by cell homing: a proof of concept study. Lancet..

[55] Kokai LE, Rubin JP, Marra KG (2005). The potential of adipose-derived adult stem cells as a source of neuronal progenitor cells. Plast Reconstr Surg.

[56] Steward AJ, Thorpe SD, Vinardell T, Buckley CT, Wagner DR, Kelly DJ (2012). Cell-matrix interactions regulate mesenchymal stem cell response to hydrostatic pressure. Acta Biomater.

[57] Jahan E, Matsumoto A, Rafiq AM, Hashimoto R, Inoue T, Udagawa J (2014). Fetal jaw movement affects Ihh signaling in mandibular condylar cartilage development: the possible role of Ihh as mechanotransduction mediator. Arch Oral Biol.

[58] Kaul R, O'Brien MH, Dutra E, Lima A, Utreja A, Yadav S (2016). The effect of altered loading on mandibular condylar cartilage. PLoS One.

[59] Heldin CH, Moustakas A (2012). Role of Smads in TGFbeta signaling. Cell Tissue Res.

[60] Li J, Wang J, Zou Y, Zhang Y, Long D, Lei L (2012). The influence of delayed compressive stress on TGF-beta1-induced chondrogenic differentiation of rat BMSCs through Smad-dependent and Smad-independent pathways. Biomaterials..

[61] Luo L, Thorpe SD, Buckley CT, Kelly DJ (2015). The effects of dynamic compression on the development of cartilage grafts engineered using bone marrow and infrapatellar fat pad derived stem cells. Biomed Mater.

[62] Connelly JT, Vanderploeg EJ, Mouw JK, Wilson CG, Levenston ME (2010). Tensile loading modulates bone marrow stromal cell differentiation and the development of engineered fibrocartilage constructs. Tissue Eng Part A.

[63] Zeiter S, Lezuo P, Ito K (2009). Effect of TGF beta1, BMP-2 and hydraulic pressure on chondrogenic differentiation of bovine bone marrow mesenchymal stromal cells. Biorheology..

[64] Huang CY, Hagar KL, Frost LE, Sun Y, Cheung HS (2004). Effects of cyclic compressive loading on chondrogenesis of rabbit bone-marrow derived mesenchymal stem cells. Stem Cells.

[65] Mauck RL, Byers BA, Yuan X, Tuan RS (2007). Regulation of cartilaginous ECM gene transcription by chondrocytes and MSCs in 3D culture in response to dynamic loading. Biomech Model Mechanobiol.

[66] Haudenschild AK, Hsieh AH, Kapila S, Lotz JC (2009). Pressure and distortion regulate human mesenchymal stem cell gene expression. Ann Biomed Eng.

[67] Fuhrer R, Hofmann S, Hild N, Vetsch JR, Herrmann IK, Grass RN (2013). Pressureless mechanical induction of stem cell differentiation is dose and frequency dependent. PLoS One.

