# Supplementary figures and images for: A novel construct with biomechanical flexibility for articular cartilage regeneration
Source: Stem Cell Res Ther. 2019 Sep 23;10:298. doi: 10.1186/s13287-019-1399-2 (PMC6757433; doi:10.1186/s13287-019-1399-2)

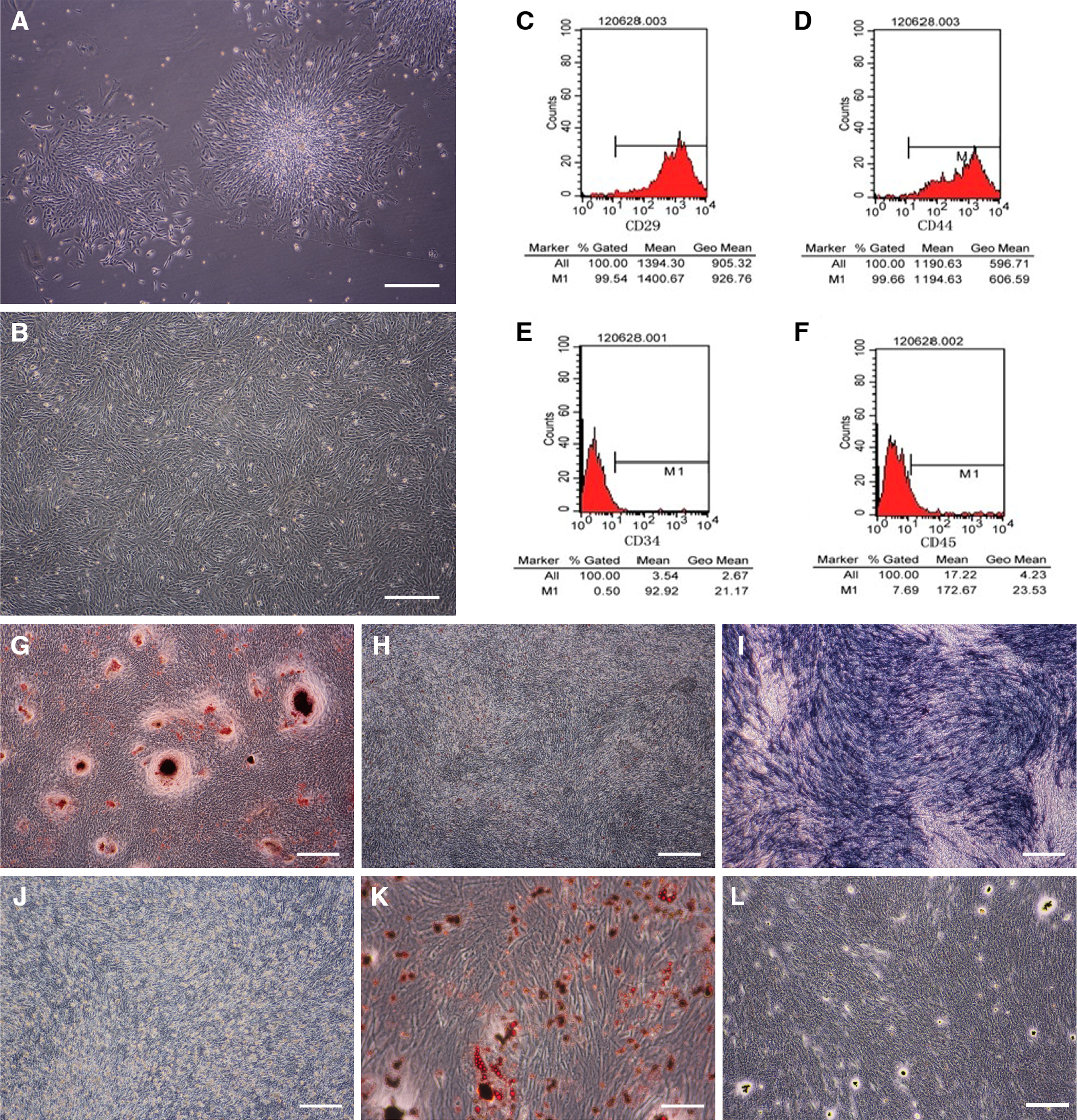

Supplement: Supplementary file 1 — Figure S1. Isolation and identification of BMSCs. (A) Cells grew rapidly, and small colonies gradually merged, with radial growth in primary culture at 7–9 days (× 40, bar = 200 μm). (B) The third generation of cells were long, fusiform, and densely arranged, in a swirl pattern (× 40, bar = 200 μm). (C-F) Flow cytometry indicated that the BMSCs were negative for hematopoietic markers CD34 and CD45 but positive for mesenchyme-associated markers CD29 and CD44. Representative figures showing the multi-directional differentiation of PDLSCs. (G) Mineralized nodules were formed after 4 weeks of osteogenic induction (stained with alizarin red, (× 40, bar = 200 μm). (I) Alkaline phosphatase staining showed a large number of blue metachromatic regions. (K) Lipid vacuoles were observed after 2 weeks of adipogenic induction (stained with oil red O, (× 40, bar = 200 μm). (H, J and L) Uninduced control cells were negative for alizarin red, alkaline phosphatase, and oil red O staining. (TIF 14270 kb) [file 13287_2019_1399_MOESM1_ESM.tif]

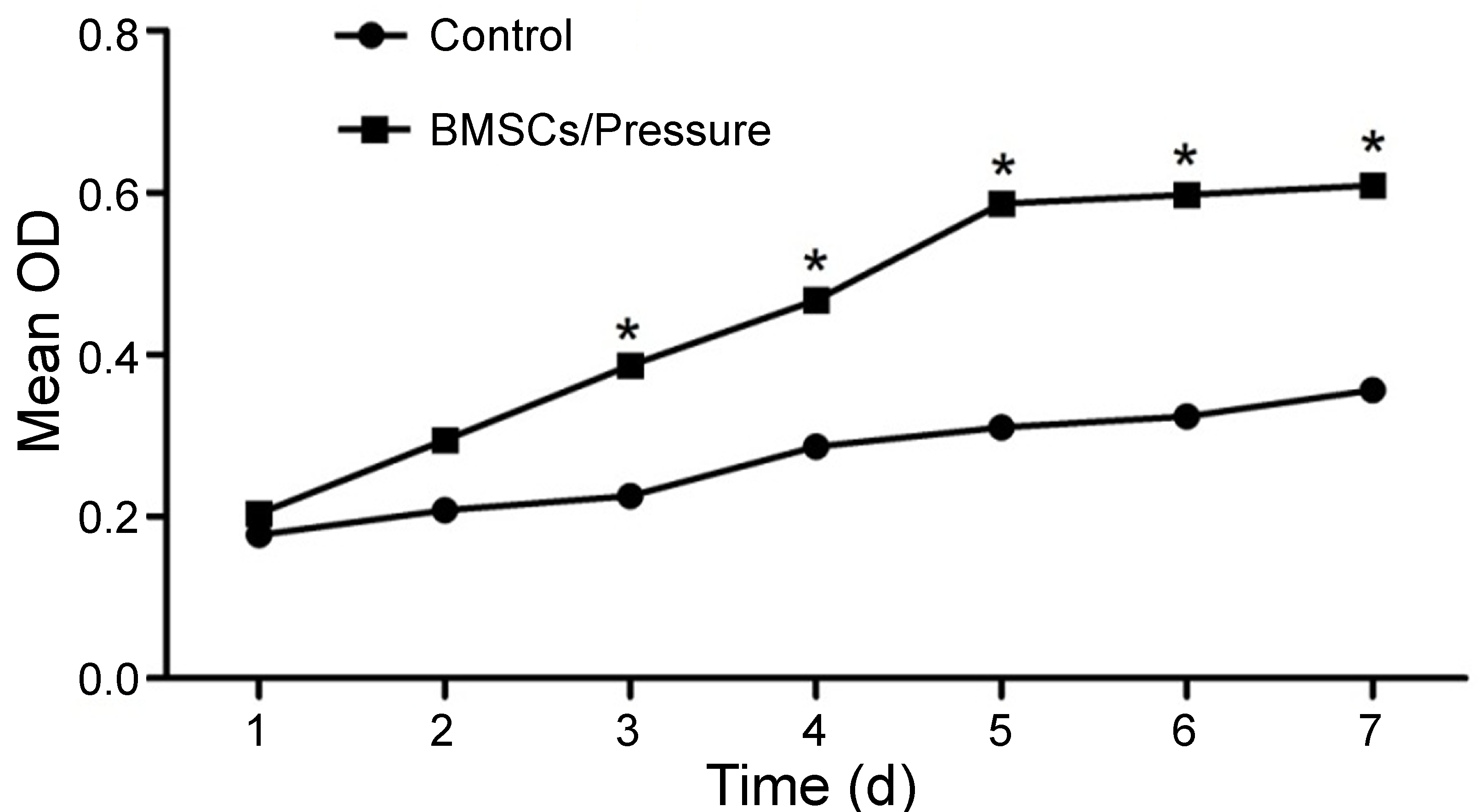

Supplement: Supplementary file 2 — Figure S2. Growth curve of BMSCs in the BMSC/PRF construct (*P < 0.05, vs. control group). (TIF 422 kb) [file 13287_2019_1399_MOESM2_ESM.tif]

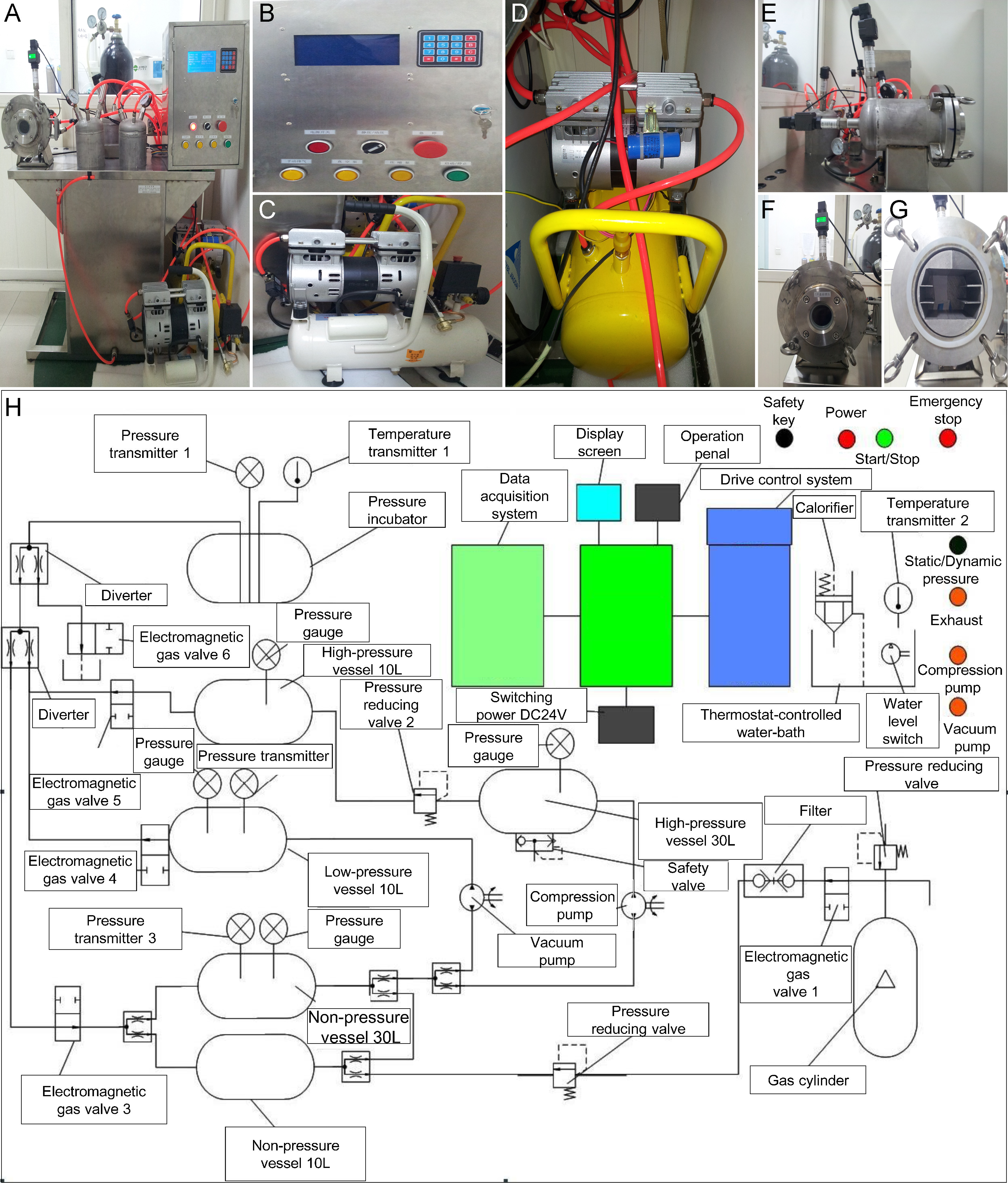

Supplement: Supplementary file 4 — Figure S3. The multi-functional pressure loading system for in vitro cultured cells. (A) Overall view of the system. (B) Control panel. (C) Drive control system–compression pump. (D) Drive control system–vacuum pump. (E) Lateral view of the pressure incubator. (F) Front view of the pressure incubator. (G) Interior of the pressure incubator. (H) Schematic illustration of the whole system. (TIF 8531 kb) [file 13287_2019_1399_MOESM4_ESM.tif]

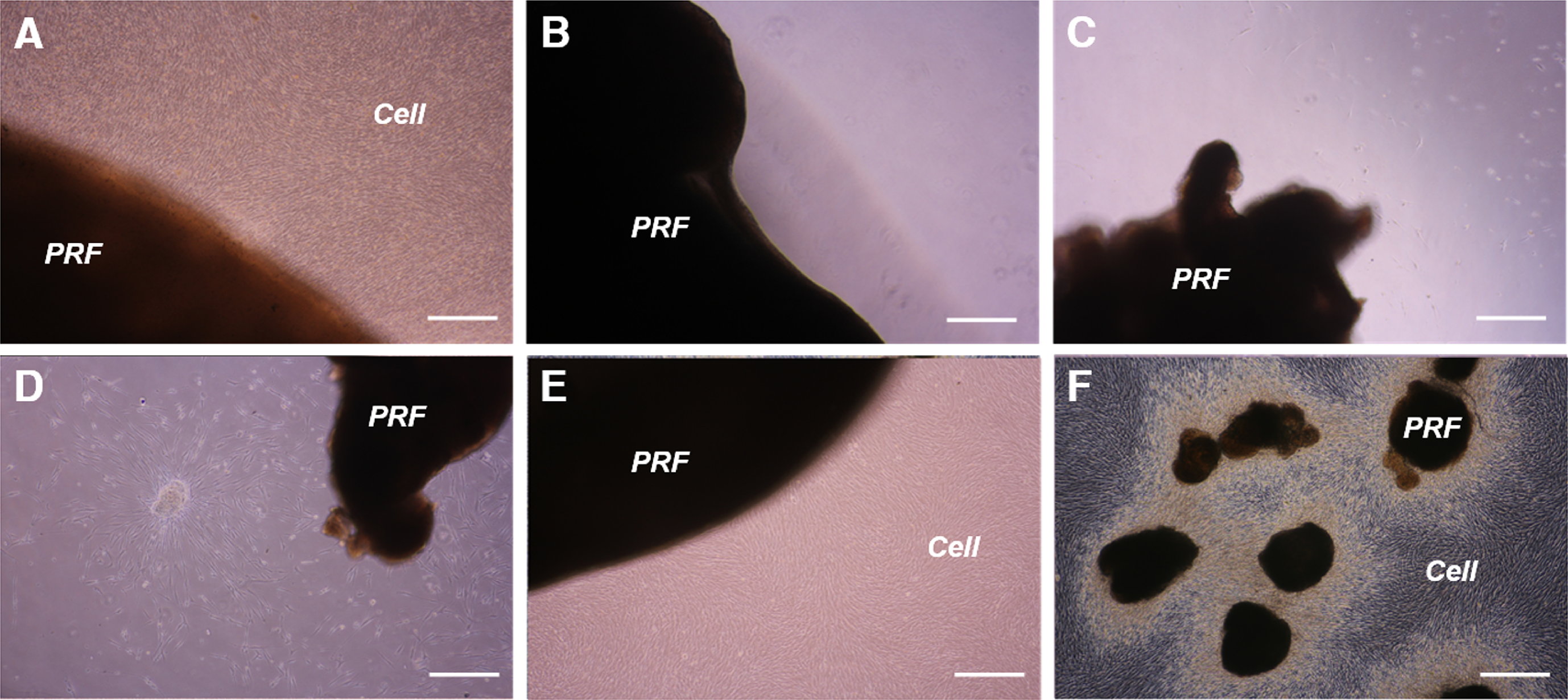

Supplement: Supplementary file 6 — Figure S4. Construction of the stem cell/PRF construct (inverted phase contrast microscopy) Group I: The PRF membrane was partially suspended. Only the thinner edge was slightly adhered to the bottom of the plate, while the thicker portion was suspended. No obvious cell proliferation was observed around the PRF membrane. Many red corpuscles were suspended in the culture medium (Figure S4A). Group II: Under the microscope, the cell sheet wrapping the PRF fragments formed an opaque construct (Figure S4B). Group III: Under the microscope, the cell sheet wrapping the PRF fragments formed an opaque construct as Group II. Group IV: The PRF granules and cell sheet fragments were mechanically embedded into each other and integrally suspended in the culture medium (Figure S4C). A small number of cell sheet fragments were scattered at the bottom of the culture plate. The latter could re-colonize at the bottom of the plate, and there were cells migrating from its edges, showing radial proliferation (Figure S4D). Group V: The PRF membrane did not adhere well to the bottom of the plate. No aggregative cell growth was observed at the edge of the PRF membrane. The PRF membrane was shaking with the culture broth (Figure S4E).;Group VI: Most PRF granules were colonized at the bottom of the Petri dish. Numerous cells showed intensive radial growth in interleaved multi-layers around the PRF granules. The cell density was higher than that in the peripheral PRF-free region (Figure S4F). (TIF 4296 kb) [file 13287_2019_1399_MOESM6_ESM.tif]

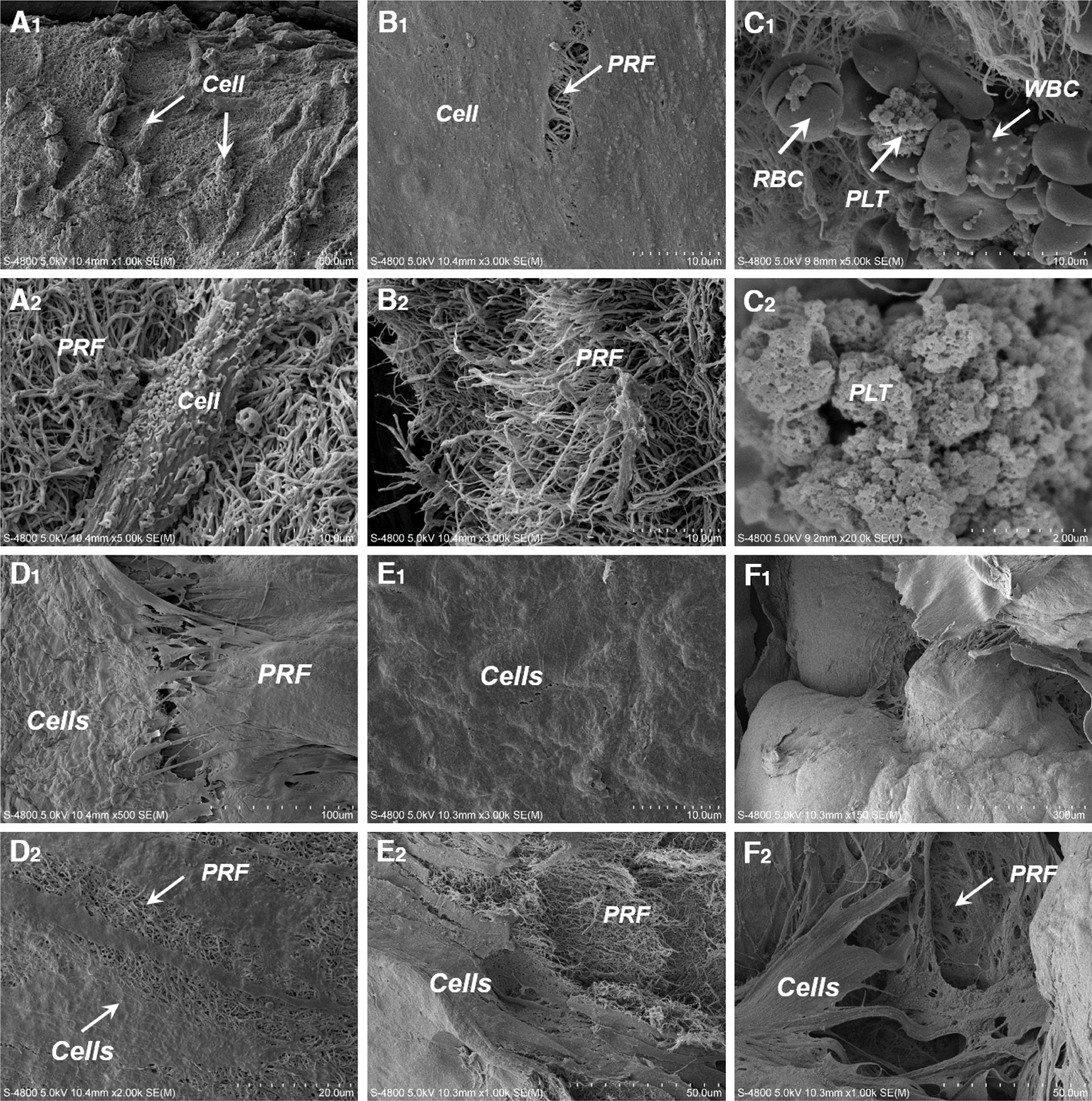

Supplement: Supplementary file 7 — Figure S5. SEM images of the construct in each group. (A1, A2) Group I. (B1, B2, C1, C2) Groups II and III. (D1, D2) Group IV. (E1, E2) Group V. (F1, F2) Group VI. Group I: The PRF structure remained in most areas of the sample. Cell structure was only observed at the thinner edge of the PRF adhered to the bottom of the plate. (Figure S5A1). Only small amount of cells became flat and evenly laid on the PRF surface; however, there was less ECM on the cell surface (Figure S5A2). Groups II and III: The cell sheet was observed retaining an intact structure when it was co-cultured with the PRF by wrapping it (Figure S5B1). In the cross-section, only the three-dimensional reticular, crosslinked structure of the PRF was observed, in the absence of any cell structure (Figure S5B2). In a few cross-sections, aggregation of red and white corpuscles (Figure S5C1) and platelets (Figure S5C2) was observed in the red end of the PRF, namely the fibrous scaffold. Group IV: In the cell sheet fragment/PRF granule co-culture group, the cell sheet was mostly curled, and a large amount of ECM (white arrow) was present on the cell surface. In the cell sheet, cells extended numerous synapses to the surface and reticular structure voids of the PRF (Figure S5D1, 2). Group V: The PRF surface was completely covered by a sheet-like cell layer after the two inoculations (Figure S5E1). No extension of cell structure was observed into the reticular voids (Figure S5E2). Group VI: In this group, the entire surface of the granular PRF was almost covered by the sheet-like structure of cells (Figure S5F1). Cell pseudopodia extended from the surface of different PRF granules and connected to the surface of other PRF granules (Figure S5F2). (TIF 10744 kb) [file 13287_2019_1399_MOESM7_ESM.tif]

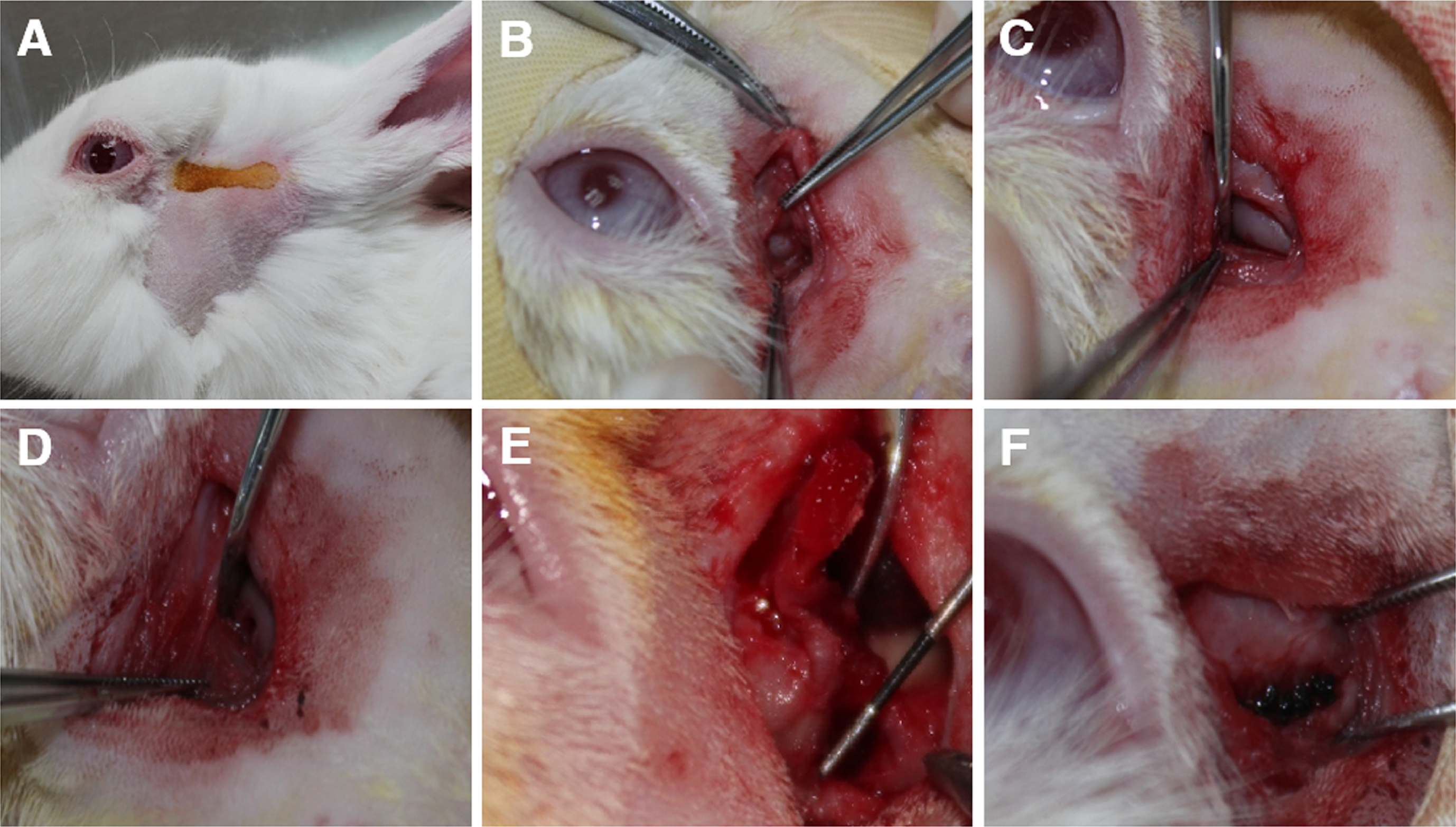

Supplement: Supplementary file 8 — Figure S6. Establishment of a cartilage defect model in bilateral temporomandibular joints. (A) An approximately 2-cm-long skin incision was made from 5 mm outside the outer canthus to the external auditory canal. (B) The joint capsule was cut open. (C) The condylar articular surface was exposed. (D, E) an approximately 3-mm-diameter hole was drilled in the center of the anterior condyle incline. (F) The joint capsule was sutured. (TIF 4677 kb) [file 13287_2019_1399_MOESM8_ESM.tif]

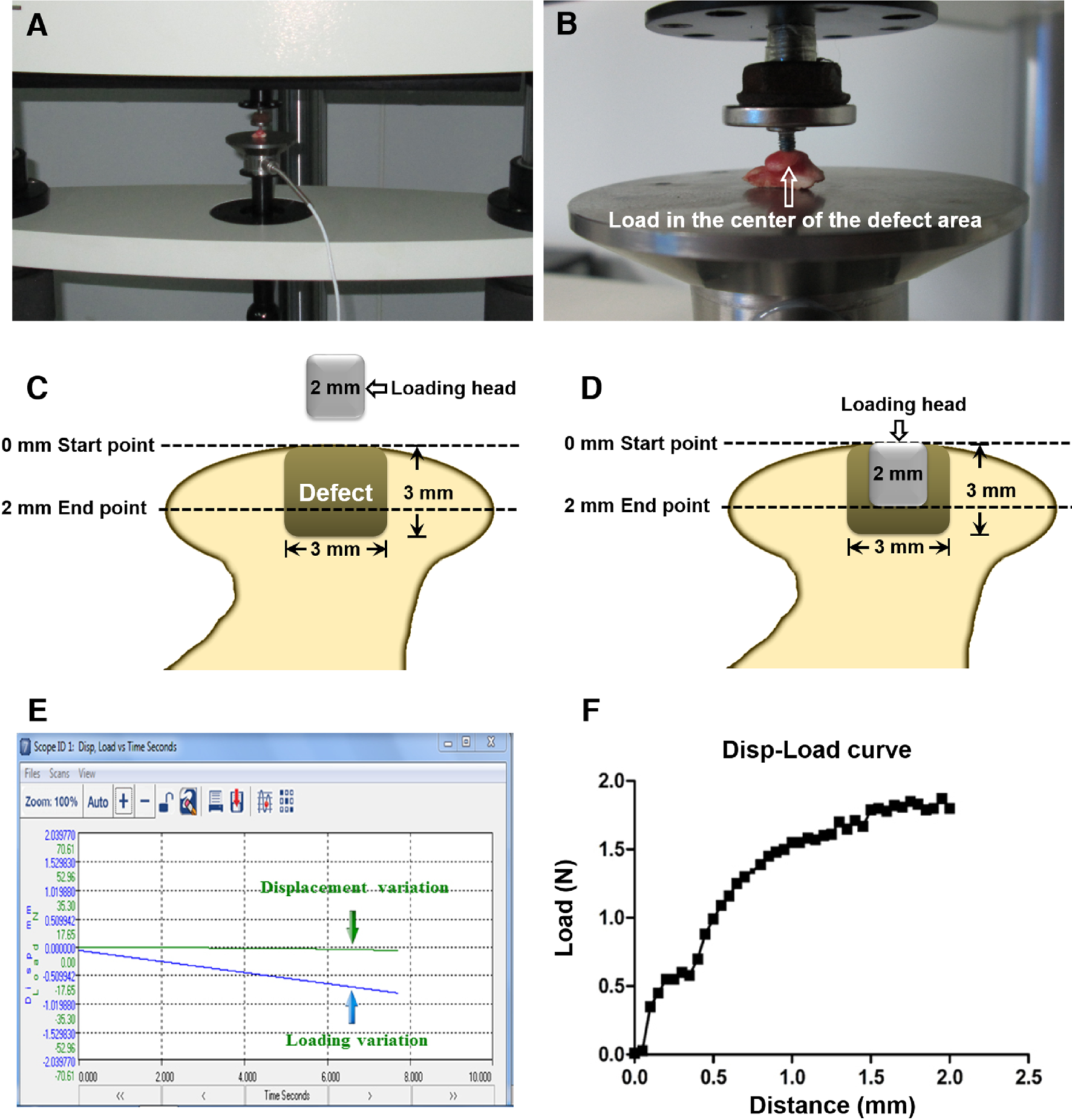

Supplement: Supplementary file 10 — Figure S8. The test instrument was applied for low-force testing of the mandibular condylar cartilage. For the biomechanical assay of the elastic modulus of the tissue-engineered cartilage, we prepared each group of condyles into a cubic shape with smooth and parallel upper and lower surfaces (Figure S8A, B). The ElectroForce® 3200 Series III test instrument (Bose Corporation Endura TEC Systems Group, Minnetonka, MN, USA) was applied for low-force testing of the engineered cartilage. We measured and calculated the cross-sectional area and height of the sample before determining the longitudinal elastic modulus. The line of force of the loading device was set perpendicular to the sample surface before measurement (Figure S8C, D). The ElectroForce® 3200 Series III test instrument (Bose Corporation Endura TEC Systems Group, Minnetonka, MN, USA) was applied for low-force testing of the engineered cartilage. The relative displacement and load were measured at a compression rate of 1 mm/min. The load–displacement curve was drawn with displacement as the abscissa axis and load as the vertical axis (Figure S8E). The elastic modulus (E) of the neocartilage was calculated according to the measured load–displacement curve (Figure S8F). (TIF 1780 kb) [file 13287_2019_1399_MOESM10_ESM.tif]
