# Supplementary material for: A novel construct with biomechanical flexibility for articular cartilage regeneration
Source: Stem Cell Res Ther. 2019 Sep 23;10:298. doi: 10.1186/s13287-019-1399-2 (PMC6757433; doi:10.1186/s13287-019-1399-2)
Supplement: Supplementary file 3 — Table S1. Detection of continued growth factor release from PRF in the BMSC/PRF construct over 96 h. (DOCX 14 kb) [file 13287_2019_1399_MOESM3_ESM.docx]

**Table S1. Detection of continued growth factor release from PRF in the BMSCs/PRF construct over 96 h**

|  | | | TGF-β1  (pg/10 ml) | | IGF-1  (pg/10 ml) | | | VEGF  (pg/10 ml) | | | EGF  (pg/10 ml) |
| --- | --- | --- | --- | --- | --- | --- | --- | --- | --- | --- | --- |
| Time | |  | |  | |  | | | |  | |
|  | 0-24 h | 3358.21 ± 21.22 | | 5528.64 ± 78.83 | | | 401.48 ± 4.31 | | 906.85 ± 5.72 | | |
|  | 24-48 h | 2518.33 ± 29.20 | | 7752.36 ± 62.24 | | | 233.87 ± 7.04 | | 825.46 ± 4.32 | | |
|  | 48-72 h | 1135.79 ± 9.19 | | 6209.81 ± 64.23 | | | 102.27 ± 5.31 | | 760.46 ± 5.18 | | |
|  | 72-96 h | 1382.99 ± 11.73 | | 4432.40 ± 57.63 | | | 89.71 ± 4.57 | | 548.90 ± 4.09 | | |
|  |  |  | |  | |  | | | |  | |
