# Supplementary material for: A novel construct with biomechanical flexibility for articular cartilage regeneration
Source: Stem Cell Res Ther. 2019 Sep 23;10:298. doi: 10.1186/s13287-019-1399-2 (PMC6757433; doi:10.1186/s13287-019-1399-2)
Supplement: Supplementary file 5 — Table S2. Primers used in Real-time PCR. (DOCX 13 kb) [file 13287_2019_1399_MOESM5_ESM.docx]

**Table S2. Primers used in Real-time polymerase chain reaction (Real-time PCR).**

| Gene | Forward primer sequence (5’-3’) | Reverse primer sequence (5’-3’) |
| --- | --- | --- |
| *Gapdh* | AGAGCACCAGAGGAGGACGA | TGGGATGGAAACTGTGAAGAGG |
| *Pcna* | GCAGGAGGGAATCCAGTTTGT | TCTCTTTTTCAGAATCTTTAGGGG |
| *Aggrecan* | GACAGAACTTTGGTAGAATCCGTAAC | ACTTGGGTCCAGAAATCCAGAAT |
| *Sox-9* | GGAGGAAGTCGGTGAAGAATGG | GGGAGTGGTGGGTGGGGT |
| *Col-II* | GCTCCCAGAACATCACCTACCAC | CAGTCTTGCCCCACTTACCG |
| *p65* | CCGCTCGAGGTATGGACGAACTGTTCC | GGGGTACCTTAGGAGCTGATCTGACTC |
