# Supplementary material for: A novel construct with biomechanical flexibility for articular cartilage regeneration
Source: Stem Cell Res Ther. 2019 Sep 23;10:298. doi: 10.1186/s13287-019-1399-2 (PMC6757433; doi:10.1186/s13287-019-1399-2)
Supplement: Supplementary file 9 — Table S3. Criteria for semi-quantitative histological scoring of articular cartilage. (DOCX 14 kb) [file 13287_2019_1399_MOESM9_ESM.docx]

**Table S3. Criteria for semi-quantitative histological scoring of articular cartilage**

| Item | Score |
| --- | --- |
| 1 Cell morphology |  |
| Normal | 4 |
| Mainly fibrocartilage | 3 |
| Mainly hyaline cartilage | 2 |
| Mainly non-cartilage | 1 |
| No cartilage | 0 |
| 2 Matrix staining (metachromasia) |  |
| Normal | 4 |
| Slightly weak  Weak | 3  2 |
| Significantly weak | 1 |
| No metachromasia | 0 |
| 3 Subchondral bone remodeling |  |
| Normal | 3 |
| Less than normal | 2 |
| Little | 1 |
| None | 0 |
| 4 Defect filling rate |  |
| 100% | 2 |
| 50%–100% | 1 |
| 0–50% | 0 |
| 5 Surface integrity* |  |
| Smooth | 1 |
| Irregular | 0 |
| 6 Cartilage thickness* |  |
| >2/3 | 2 |
| 1/3–2/3 | 1 |
| <1/3 | 0 |
| 7 Connection |  |
| Binding on both sides | 2 |
| Binding on one side | 1 |
| No bonding | 0 |

Note: * comparison between the mean thickness of cartilage tissue in the repair region and the thickness of surrounding normal cartilage tissue; maximum score = 18 points.
